# Impact of critical eddy diffusivity on seasonal bloom dynamics of Phytoplankton in a global set of aquatic environments

**DOI:** 10.1038/s41598-023-43745-z

**Published:** 2023-10-10

**Authors:** Arpita Mondal, Sandip Banerjee

**Affiliations:** Department of Mathematics, Indian Institute of Technology Roorkee, Roorkee, Uttarakhand 247667 India

**Keywords:** Ecological modelling, Theoretical ecology, Marine biology

## Abstract

The intensity of eddy diffusivity and the spatial average of water velocity at the depths of the water column in oceans and lakes play a fundamental role in phytoplankton production and phytoplankton and zooplankton biomass, and community composition. The critical depth and intensity of turbulent mixing within the water column profoundly affect phytoplankton biomass, which depends on the sinking characteristic of planktonic algal species. We propose an Nutrient-Phytoplankton-Zooplankton (NPZ) model in 3D space with light and nutrient-limited growth in a micro-scale ecological study. To incorporate micro-scale observation of phytoplankton intermittency in bloom mechanism in stationary as well as oceanic turbulent flows, a moment closure method has been applied in this study. Experimental observations imply that an increase in turbulence is sometimes ecologically advantageous for non-motile planktonic algae. How do we ensure whether there will be a bloom cycle or whether there can be any bloom at all when the existing phytoplankton group is buoyant, heavier, motile, or non-motile? To address these questions, we have explored the effects of critical depth, the intensity of eddy diffusivity, spatial average of water velocity, on the concentration as well as horizontal and vertical distribution of phytoplankton and zooplankton biomass using a mathematical model and moment closure technique. We quantify a critical threshold value of eddy diffusivity and the spatial average of water velocity and observe the corresponding changes in the phytoplankton bloom dynamics. Our results highlight the importance of eddy diffusivity and the spatial average of water velocity on seasonal bloom dynamics and also mimic different real-life bloom scenarios in Mikawa Bay (Japan), Tokyo Bay (Japan), Arakawa River (Japan), the Baltic Sea, the North Atlantic Ocean, Gulf Alaska, the North Arabian Sea, the Cantabrian Sea, Lake Nieuwe Meer (Netherlands) and several shallower lakes.

## Introduction

The impact of the physical mixing process on the interaction of phytoplankton species is immense^1–3^. The occurrence of phytoplankton bloom gets triggered by a rapid increment in the spatial distribution of phytoplankton density. Fundamentally speaking, regional and seasonal changes influence environmental factors like solar irradiance and water salinity, which cause irregularities in phytoplankton growth. The nature of dominating or existing plankton classes changes with regional and seasonal changes. Different phytoplankton classes possess different traits, most notably size, shape, specific rate of growth, and behavior, such as the nature of floating or sinking, which all together determine their dynamical behavior in an aquatic ecosystem.

Eddy diffusion exerts an intriguing impact on the spatial distribution of plankton species along horizontal and vertical directions for any turbulent aquatic environment^4^. Changes in turbulent mixing cause a significant shift in the existing phytoplankton community. Increase in the oceanic mixed layer depth negatively affects the leading light available to planktonic algae and the sedimentation loss rates of sinking phytoplankton, resulting in an overall decrease in phytoplankton growth with increasing mixing depth^5^. While buoyant species mostly float upwards during weak mixing, intense mixing causes species to dive into a deeper zone. However, significant change in the intensity of vertical mixing triggers the transportation of nutrients and other essential ingredients required for photosynthesis from the surface layer to a higher depth. Nutrient enrichment positively affects nutrient availability and phytoplankton growth, resulting in persistence of sinking species into deeper zones, which initiates the chances of bloom development at higher depths. The nature of bloom gets dominated by the nature of mixing, which varies inversely with surface water density and stability. When it comes to the density of surface water, variation in temperature, humidity, and salinity play a role, and these components vary with seasonal and regional changes, as a result of which, intensity of turbulent mixing varies along with the nature of bloom gets decided. So, the role of vertical eddy diffusivity is quite important in bloom development at higher depths.

The other two factors that play essential roles in bloom initiation, either on the surface layer or at higher depths, are the swimming and sinking velocity of existing species, which get decided by the nature of the existing class. Generally, in the presence of weak mixing, if the swimming velocity of the existing community dominates over sinking velocity, then the existing community floats away instead of diving into a deeper zone; such circumstances trigger the chances of surface bloom. On the other hand, if existing species have a sinking tendency, then generally, bloom occurs at higher depth, which has been observed for several species on a global scale^4^.

The biological interactions between oceanic populations in turbulent flows drive plankton dynamics and cycling of nutrients, with impacts expected to be accumulative across multiple scales^6^. Measurements of phytoplankton distributions have improved with the advancement of modern technologies. Considering recent observations at finer resolution, the in situ spatial patterns become increasingly important for understanding the mechanisms that create and support the micro-scale structure and ecology of marine ecosystems^7^.

The influence of both physical and biological processes on phytoplankton dynamics from mesoscale down to microscale, as revealed by the high-resolution instruments, must be considered for a mechanistic understanding of plankton ecosystems. Therefore, further theoretical developments and new modelling tools are required to understand the observed small-scale vertical structure and its relationship to ecosystem behaviour. Intense intermittency in plankton distributional dynamics is observed pervasively in marine ecology, mostly when high-resolution instruments such as microstructure profiling fluorometers, water samplings, FIDO-$\phi$^7,8^ are capable of measuring high-resolution data of micro-scale planktonic ecosystem. Such advancement in technologies capture acute dynamical phenomena and in fact, this measurement has exhibited that spatial variability becomes rapidly irregular as we move from meso-scale to micro-scale distribution. Under such circumstances, inclusion of moment closure methodology on ecological modeling dynamics helps in a better understanding of phytoplankton bloom mechanism, since it captures dynamical nonlinearity acting on spatial variability of micro-scale observation.

A conventional study with closure approach can provide some dynamical understanding of phytoplankton bloom dynamics, still certain unresolved queries sustain, for example, how the NPZ closure model behaves with physics (critical depth, eddy diffusivity, water velocity) in a turbulent medium in comparison with the conventional NPZ model. To understand the impact of physics on dynamical behaviour of NPZ ecosystem, we have explored the effects of critical depth, the intensity of eddy diffusivity, spatial average of water velocity, on the concentration as well as horizontal and vertical distribution of phytoplankton and zooplankton biomass using a 3D biophysical NPZ closure model to visualize the impact of sub-scale variability coupled with physical transport.

Our study is designed to exhibit mathematical facts to illustrate the bloom cycle, surface bloom, bloom in higher depth, and no bloom at all, for micro-scale dynamics in both turbulent and stationary aquatic environments. The goal of this study is to provide a mathematical understanding behind all possible patterns of seasonal bloom dynamics based on a newly developed mathematical understanding in the micro-scale analysis, for example, why the surface bloom of any phytoplankton species, whether buoyant or heavier, occurs during the wind mixing period in the warmer season without any artificial mixing; why bloom in higher depths is most common in winter, without wind mixing; what triggers red-tide formation in the windy zone and, what is the impact of artificial mixing in bloom dynamics for low grazing zones? Our results highlight the importance of eddy diffusivity and the spatial average of water velocity on seasonal bloom dynamics and also mimic different real-life bloom scenarios in Mikawa Bay (Japan), Tokyo Bay (Japan), Arakawa river (Japan), the Baltic sea, the North Atlantic Ocean, Gulf Alaska, the North Arabian Sea, the Cantabrian Sea, Lake Nieuwe Meer (Netherlands) and several shallower lakes^8–16^.

## Materials and methods

### Model definition: conventional nutrient-phytoplankton-zooplankton (NPZ) model

According to several observations regarding phytoplankton bloom occurrences in a global set of aquatic environments (Table 1), we initially propose an NPZ model in 3D space considering the contribution of turbulence mixing and the nature of phytoplankton species, where we have later applied the moment closure method for better understanding of bloom mechanism. Starting with non-averaged small-scale equations and ignoring molecular and turbulent diffusion, we first consider the space-time evolution equation for the NPZ ecosystem model, given by

1 $$\begin{aligned} \frac{\partial N}{\partial t}=&-\frac{\partial }{\partial x}(u+k_{n}) {N}-\frac{\partial }{\partial y}(v+k_{n}) {N}-\frac{\partial }{\partial z}(w+w_{n}){N}+S_{1}(N, P, Z), \end{aligned}$$

2 $$\begin{aligned} \frac{\partial P}{\partial t}=&-\frac{\partial }{\partial x}(u+k_{p}) {P}-\frac{\partial }{\partial y}(v+k_{p}) {P}-\frac{\partial }{\partial z}(w+w_{p}){P}+S_{2}(N, P, Z), \end{aligned}$$

3 $$\begin{aligned} \frac{\partial Z}{\partial t}=&-\frac{\partial }{\partial x}(u+k_{z}) {Z}-\frac{\partial }{\partial y}(v+k_{z}) {Z}-\frac{\partial }{\partial z}(w+w_{z}){Z}+S_{3}(N, P, Z). \end{aligned}$$

In this model, u, v are the horizontal water velocities, w is the vertical water velocity, ${k_n}, k_{p}, {k_z}$ are the horizontal swimming speed of nutrient, phytoplankton and zooplankton, ${w_n},w_{p},{w_z}$ are the vertical sinking velocity of nutrient, phytoplankton, and zooplankton respectively, and (x, y, z) is the spatial coordinate vector. Here, we have assumed, ${w_n}={k_n}={w_z}={k_z}=0$, whereas ${k_p}\ne 0,{w_p}\ne 0$. $S_{i}(N,P,Z)\,(i=1,2,3)$ gives the bio-physical interaction terms among state variables *N*, *P*, *Z* describing a three dimensional aquatic ecosystem, given by

$$\begin{aligned} S_{1}(N, P, Z)=&-\mu _{p}\,P+(m_{P}+r_P+s+k_{2})+(1-a_{z}){g_z}PZ+(\tau _{z}+{m_{z}}Z)Z,\\ S_{2}(N, P, Z)=&\mu _{p}\,P-(m_{P}+r_P+s+k_{2})\,P-g_{z}P\,Z,\\ S_{3}(N, P, Z)=&a_{z}{g_z}PZ-(\tau _{z}+{m_{z}}Z)\,Z, \end{aligned}$$

with

$$\begin{aligned}&\sum _{i=1}^{3} S_{i}(N,P,Z)=0\Rightarrow \frac{dN}{dt}+\frac{dP}{dt}+\frac{dZ}{dt}=0\Rightarrow N+P+Z=A\,(\text {constant}), \end{aligned}$$

which indicates that the corresponding bio-physical ODE system is closed and conserved. Here, $\mu _{p}=g(I)F(N, P)$ indicates phytoplankton growth term including two types of growth, namely,

(i) light limited growth indicated by term $g(I)=\frac{V_{m}\alpha I}{({V_m}^2+(\alpha I)^2)^{\frac{1}{2}}}$, where $I={I_0}e^{-\kappa H}$, $I_0$ is surface irradiance, $\kappa$ is light attenuation coefficient and *H* is the depth of the layer within the water column at which irradiance *I* is measured^17^,
(ii) nutrient limited growth, indicated by Holling type II functional response, $F(N, P)=\frac{N}{K+N}$.

The term $(m_{P}+r_P+s+k_{2})$ describes the overall phytoplankton loss due to normal mortality ($m_P$), respiration ($r_P$), sinking (*s*) and cross thermocline exchange ($k_2$). The term $g_{z}PZ$ is the loss of phytoplankton due to zooplankton grazing, where $g_z$ is the grazing rate. Suppose $a_z$ is the food conversion efficiency of zooplankton. In that case, zooplankton growth includes $a_{z}g_{z}PZ$ caused by assimilated grazing impact, whereas zooplankton loss includes excretion ($\tau _z\,Z$) and mortality due to competition (${m_z}Z^2$). As the proposed system is conserved, there is no external source for nutrient *N*. The growth of phytoplankton causes nutrient loss, and the system regains nutrients from the loss of phytoplankton and zooplankton.

**Table 1:** Global observation: bloom dynamics.

Location	Time	Nature of bloom/density of species	Species/community	External factor
Tokyo Bay^8^	Dec, 2006	Bloom at 50 meter	*Skeletonema Costatum*	Variation in temperture
	Feb, 2008			
Arakawa River, Tokyo^8^	May, 2011	Bloom at upper surface	*Skeletonema Costatum*	Variation in temperature
North Arabian Sea^13^	Jan–March	Deep sea water	Algae	Variation in temperature
Mikawa Bay, Japan^9^	Spring, 1991	Surface bloom	*Alexandrium Tamarense*	Diffusion, upwelling
Mikawa Bay, Japan^9^	July, 1990 (Summer)	Surface bloom	*Ciliate*	Diffusion, upwelling
Mikawa Bay, Japan^9^	Nov, 1991 (Autumn)	Surface bloom	*Mesodinium Rubrum*	Diffusion, upwelling
Baltic Sea^10^	Feb/March	Surface bloom	Dinoflagelates/Diatom	Warming of surface water
Mikawa Bay, Japan^9^	1990:11–20 June, 17–25 June, 1990: 11–16 July,13–17 July 1990: 11–20 Aug, 11–23 Aug 1990:22–29 Sept, 2–8 Oct 1991:1–5Mar, 10–17 Apr 1991:20–27 June, 29 July–2 Aug 1991:21–30 Sep	Bloom cycle	*Skeletonema Costatum*	Wind mixing and thermal stratification of water
Lake Nuewe Meer^15^	Aug, 2002 (some days)	Bloom at higher depth	*Microcystis*	Artificial mixing
Temperate North Atlantic Ocean^11^	Spring, 2018	Surface bloom	Chl-a	Atmospheric forcing, strong wind
Central Cantabrian Sea^14^	Late Winter	Surface bloom	Chl-a, Chl-b Chl-c	Slight increment in water temperature
Gulf Alaska^12^	2014–2016	Low density on surface layer	Diatom	Marine heat wave, Higher grazing
Gulf Alaska^12^	2014–2016	High density	Zooplankton	Marine heat wave, High grazing
Shallow lakes^16^	Spring season	Surface bloom	Diatom	Temperate water
Shallow lakes^16^	Spring season	Abundance on surface water	Copepods (* Daphnia hyalina-galeata *)	Temperate water

### NPZ closure model

During the studies of the distribution of microscale phytoplankton using high-resolution profiling fluorometers, it has been observed that the local fluorescence values are highly fluctuating in space^7,18^. Since the density of zooplankton is interrelated to the density of phytoplankton and phytoplankton growth is interconnected to the density of nutrients available in the closed environment, all three variables *N*, *P*, *Z* are expected to be fluctuating in space in the closed aquatic ecosystem. However, the non-closure model as described by Eqs. (1)–(3) does not consider this spatial variability. To derive the NPZ-closure model from the conventional NPZ model (non-closure), the model variables are considered to be a function of both time (t) and space (r), namely,

4 $$\begin{aligned}&N(r,t)=N_{0}(r,t)+ N^{'}(r,t),\,P(r,t)=P_{0}(r,t)+ {P'}(r,t)\,\text {and}\,Z(r,t)=Z_{0}(r,t)+ {Z'}(r,t), \end{aligned}$$

where $N_{0}$, $P_{0}, Z_{0}$ are spatial mean values of the nutrient, phytoplankton, and zooplankton communities respectively, and $N^{'}$, ${P'}, {Z'}$ are their respective fluctuating components corresponding to each mean value. The horizontal and vertical sampling for microscale phytoplankton distribution has the same statistics at the centimeter scale^19^ and also at the millimeter scale (except for extreme values). Hence, the statistics of the fluctuating components is independent of the direction of sampling (isotropic). Therefore, the spatial average of each fluctuating component is zero at any particular time, that is, $<N^{'}(r)>$ = 0, $<{P'}(r)>$ = 0, $<{Z'}(r)>$ = 0, while its temporal average cannot be zero, which implies $<N(t)>=N_{0}(t)$, $<P (t)>=P_{0}(t)$ and $<Z(t)>=Z_{0}(t)$. Also, water velocities *u*, *v*, *w* are fluctuating components in space and time, whereas $k_{n}, {w_n}, k_{p}, {w_p}, {k_z}$ and $w_{z}$ are non fluctuating quantities, that is,

5 $$\begin{aligned}{}&u=\langle u\rangle + u',v=\langle v\rangle +v', w=\langle w\rangle +w' \,\text {with}\, \langle u'\rangle =0,\langle v'\rangle =0,\langle w'\rangle =0. \end{aligned}$$

### **Modelling framework**

Substituting (4–5) in (1), and applying the Reynold’s averaging method in space, we obtain,

$$\begin{aligned}{}&\frac{\partial }{\partial t}{N_0}+(\langle u\rangle +k_{n})\frac{\partial {N_0} }{\partial x}+\frac{\partial }{\partial x}\left( \langle u'N'\rangle \right) +(\langle v\rangle +k_{n})\frac{\partial {N_0} }{\partial y}+\frac{\partial }{\partial y}\left( \langle v'N'\rangle \right) +(\langle w\rangle +w_{n})\frac{\partial {N_0} }{\partial z}+\frac{\partial }{\partial z}\left( \langle w'N'\rangle \right) \\&\quad =\langle S_{1}({N_0}+N', {N_0}+P', {N_0}+Z')\rangle , \end{aligned}$$

since $\langle u\rangle , \langle v\rangle , \langle w\rangle , k_n, w_n$ are independent of *x*, *y*, *z*.

Three terms on the left hand side of the above equation namely, $\langle u'{N'}\rangle , \langle v'N'\rangle , \langle w'N'\rangle$, are covariance terms between velocity fluctuations and fluctuations of nutrient density, and therefore denote turbulent transports. These are classically parameterized by means of a down-gradient approach as

$$\begin{aligned}{}&\langle u'{N'}\rangle =-{k_h}\frac{\partial \langle N\rangle }{\partial x}=-{k_h}\frac{\partial {N_0} }{\partial x},\,\, \langle v'{N'}\rangle =-{k_h}\frac{\partial \langle N\rangle }{\partial y}=-{k_h}\frac{\partial {N_0} }{\partial y},\,\, \langle w'{N'}\rangle =-{k_v}\frac{\partial \langle N\rangle }{\partial z}=-{k_h}\frac{\partial {N_0} }{\partial z}, \end{aligned}$$

with horizontal and vertical eddy diffusion coefficient or turbulent diffusion coefficient or eddy diffusivity $k_h$ and $k_v$ respectively. Therefore, substituting these values of $\langle u'{N'}\rangle , \langle v'N'\rangle , \langle w'N'\rangle$, we obtain

$$\begin{aligned}{}&\frac{\partial }{\partial t}{N_0}+(\langle u\rangle +k_{n})\frac{\partial {N_0} }{\partial x}+(\langle v\rangle +k_{n})\frac{\partial {N_0} }{\partial y}+(\langle w\rangle +w_{n})\frac{\partial {N_0} }{\partial z}\\&\quad +\frac{\partial }{\partial x}\left( -{k_h}\frac{\partial {N_0} }{\partial x}\right) +\frac{\partial }{\partial y}\left( -{k_h}\frac{\partial {N_0} }{\partial y}\right) +\frac{\partial }{\partial z}\left( -{k_v}\frac{\partial {N_0} }{\partial z}\right) \\&\quad =\langle S_{1}({N_0}+N', {P_0}+P', {Z_0}+Z')\rangle . \end{aligned}$$

Considering $k_h, k_v$ to be constant parameters, we obtain,

$$\begin{aligned}{}&\frac{\partial }{\partial t}{N_0}+(\langle u\rangle +k_{n})\frac{\partial {N_0} }{\partial x}+(\langle v\rangle +k_{n})\frac{\partial {N_0} }{\partial y}+(\langle w\rangle +w_{n})\frac{\partial {N_0} }{\partial z}-{k_h}\frac{\partial ^2 {N_0}}{\partial x^2}\\&\quad -{k_h}\frac{\partial ^2 {N_0}}{\partial y^2}-{k_v}\frac{\partial ^2 {N_0}}{\partial z^2}=\langle S_{1}({N_0}+N', {P_0}+P', {Z_0}+Z')\rangle \end{aligned}$$

To construct a closure for these quantities, we follow the principle of second order turbulence closure modelling^20^ and first derive transport equations for $N', P'$ and $Z'$ by subtracting the equation for $N_0, P_0, Z_0$ from the equation for *N* and accordingly for *P* and *Z*. Once these equations are derived, we note that

6 $$\begin{aligned}{}&\langle N'\frac{\partial N'}{\partial t}\rangle =\frac{1}{2}\frac{\partial \langle N'^2 \rangle }{\partial t},\,\,\langle P'\frac{\partial P'}{\partial t}\rangle =\frac{1}{2}\frac{\partial \langle P'^2 \rangle }{\partial t},\,\,\langle Z'\frac{\partial Z'}{\partial t}\rangle =\frac{1}{2}\frac{\partial \langle Z'^2 \rangle }{\partial t},\,\,\langle P'\frac{\partial N'}{\partial t}+N'\frac{\partial P'}{\partial t}\rangle =\frac{\partial \langle N'P' \rangle }{\partial t},\nonumber \\&\langle P'\frac{\partial Z'}{\partial t}+Z'\frac{\partial P'}{\partial t}\rangle =\frac{\partial \langle P'Z' \rangle }{\partial t},\,\, \langle Z'\frac{\partial N'}{\partial t}+N'\frac{\partial Z'}{\partial t}\rangle =\frac{\partial \langle N'Z' \rangle }{\partial t}. \end{aligned}$$

The consequent application of this derivation would result in vertical turbulent flux divergence formulations for the second order moments $\langle N'^2\rangle , \langle P'^2 \rangle , \langle Z'^2 \rangle , \langle N'P' \rangle , \langle P'Z' \rangle , \langle N'Z' \rangle$, which are not in gradient form. Therefore, we ignore vertical turbulent fluxes in the derivation and later parameterize the vertical flux in the down-gradient form with the same eddy diffusivity as for the other tracers^21^, and obtain

7 $$\begin{aligned}{}&\frac{\partial N'}{\partial t}+(\langle u\rangle +{k_n})\frac{\partial N'}{\partial x}+(\langle v\rangle +{k_n})\frac{\partial N'}{\partial y}+(\langle w\rangle +{w_n})\frac{\partial N'}{\partial z}={S_1}(N, P, Z)-\langle {S_1}(N, P, Z)\rangle , \end{aligned}$$

8 $$\begin{aligned}{}&\frac{\partial P'}{\partial t}+(\langle u\rangle +{k_p})\frac{\partial P'}{\partial x}+(\langle v\rangle +{k_p})\frac{\partial P'}{\partial y}+(\langle w\rangle +{w_p})\frac{\partial P'}{\partial z}={S_2}(N, P, Z)-\langle {S_2}(N, P, Z)\rangle , \end{aligned}$$

9 $$\begin{aligned}{}&\frac{\partial Z}{\partial t}+(\langle u\rangle +{k_z})\frac{\partial Z}{\partial x}+(\langle v\rangle +{k_z})\frac{\partial Z}{\partial y}+(\langle w\rangle +{w_z})\frac{\partial Z}{\partial z}={S_3}(N, P, Z)-\langle {S_3}(N, P, Z)\rangle \end{aligned}$$

Using (6), and assuming that the variances and the covariances, $\langle P'^2 \rangle$, $\langle N'^2 \rangle$, $\langle Z'^2 \rangle$, $\langle N'P' \rangle$, $\langle P'Z' \rangle$, $\langle N'Z' \rangle$, follow the same diffusion law as $N_0, P_0, Z_0$ and assuming the covariances $\langle N'P'\rangle , \langle P'Z'\rangle , \langle N'Z'\rangle$ have horizontal and vertical settling velocity $k_{np}, k_{pz}, k_{nz}$ and $w_{np}, w_{pz}, w_{nz}$ respectively, and re-introducing vertical flux divergences for the second-moment equations, we finally arrive at the following set of equations:

10 $$\begin{aligned}{}&\frac{\partial }{\partial t}{N_0}+(\langle u\rangle +k_{n})\frac{\partial {N_0} }{\partial x}+(\langle v\rangle +k_{n})\frac{\partial {N_0} }{\partial y}+(\langle w\rangle +w_{n})\frac{\partial {N_0} }{\partial z}-{k_h}\frac{\partial ^2 {N_0}}{\partial x^2}-{k_h}\frac{\partial ^2 {N_0}}{\partial y^2}-{k_v}\frac{\partial ^2 {N_0}}{\partial z^2}\nonumber \\&\quad =\langle S_{1}({N_0}+N', {P_0}+P', {Z_0}+Z')\rangle \end{aligned}$$

11 $$\begin{aligned}{}&\frac{\partial }{\partial t}{P_0}+(\langle u\rangle +k_{n})\frac{\partial {P_0} }{\partial x}+(\langle v\rangle +k_{n})\frac{\partial {P_0} }{\partial y}+(\langle w\rangle +w_{n})\frac{\partial {P_0} }{\partial z}-{k_h}\frac{\partial ^2 {P_0}}{\partial x^2}-{k_h}\frac{\partial ^2 {P_0}}{\partial y^2}-{k_v}\frac{\partial ^2 {P_0}}{\partial z^2}\nonumber \\&\quad =\langle S_{2}({N_0}+N', {P_0}+P', {Z_0}+Z')\rangle \end{aligned}$$

12 $$\begin{aligned}{}&\frac{\partial }{\partial t}{Z_0}+(\langle u\rangle +k_{n})\frac{\partial {Z_0} }{\partial x}+(\langle v\rangle +k_{n})\frac{\partial {Z_0} }{\partial y}+(\langle w\rangle +w_{n})\frac{\partial {Z_0} }{\partial z}-{k_h}\frac{\partial ^2 {Z_0}}{\partial x^2}-{k_h}\frac{\partial ^2 {Z_0}}{\partial y^2}-{k_v}\frac{\partial ^2 {Z_0}}{\partial z^2}\nonumber \\&\quad =\langle S_{3}({N_0}+N', {P_0}+P', {Z_0}+Z')\rangle \end{aligned}$$

13 $$\begin{aligned}{}&\frac{\partial }{\partial t}{ \langle N'^2\rangle }+(\langle u\rangle +k_{n})\frac{\partial {\langle N'^2\rangle } }{\partial x}+(\langle v\rangle +k_{n})\frac{\partial {\langle N'^2\rangle } }{\partial y}+(\langle w\rangle +w_{n})\frac{\partial {\langle N'^2\rangle } }{\partial z}-{k_h}\frac{\partial ^2 {\langle N'^2\rangle }}{\partial x^2}-{k_h}\frac{\partial ^2 {\langle N'^2\rangle }}{\partial y^2}\nonumber \\&\quad -{k_v}\frac{\partial ^2 {\langle N'^2\rangle }}{\partial z^2}=2\langle N' S_{1}({N_0}+N', {P_0}+P', {Z_0}+Z')\rangle \end{aligned}$$

14 $$\begin{aligned}{}&\frac{\partial }{\partial t}{\langle P'^2\rangle }+(\langle u\rangle +k_{n})\frac{\partial {\langle P'^2\rangle } }{\partial x}+(\langle v\rangle +k_{n})\frac{\partial {\langle P'^2\rangle } }{\partial y}+(\langle w\rangle +w_{n})\frac{\partial {\langle P'^2\rangle } }{\partial z}-{k_h}\frac{\partial ^2 {\langle P'^2\rangle }}{\partial x^2}-{k_h}\frac{\partial ^2 {\langle P'^2\rangle }}{\partial y^2}\nonumber \\&\quad -{k_v}\frac{\partial ^2 {\langle P'^2\rangle }}{\partial z^2}=2\langle P' S_{2}({N_0}+N', {P_0}+P', {Z_0}+Z')\rangle \end{aligned}$$

15 $$\begin{aligned}{}&\frac{\partial }{\partial t}{\langle Z'^2\rangle }+(\langle u\rangle +k_{n})\frac{\partial {\langle Z'^2\rangle } }{\partial x}+(\langle v\rangle +k_{n})\frac{\partial {\langle Z'^2\rangle } }{\partial y}+(\langle w\rangle +w_{n})\frac{\partial {\langle Z'^2\rangle } }{\partial z}-{k_h}\frac{\partial ^2 {\langle Z'^2\rangle }}{\partial x^2}-{k_h}\frac{\partial ^2 {\langle Z'^2\rangle }}{\partial y^2}\nonumber \\&\quad -{k_v}\frac{\partial ^2 {\langle Z'^2\rangle }}{\partial z^2}=2\langle Z' S_{3}({N_0}+N', {P_0}+P', {Z_0}+Z')\rangle \end{aligned}$$

16 $$\begin{aligned}{}&\frac{\partial }{\partial t}{\langle N'P'\rangle }+(\langle u\rangle +k_{n})\frac{\partial {\langle N'P'\rangle } }{\partial x}+(\langle v\rangle +k_{n})\frac{\partial {\langle N'P'\rangle } }{\partial y}+(\langle w\rangle +w_{n})\frac{\partial {\langle N'P'\rangle } }{\partial z}-{k_h}\frac{\partial ^2 {\langle N'P'\rangle }}{\partial x^2}\nonumber \\&\quad -{k_h}\frac{\partial ^2 {\langle N'P'\rangle }}{\partial y^2} -{k_v}\frac{\partial ^2 {\langle N'P'\rangle }}{\partial z^2}=\langle P' S_{1}({N_0}+N', {P_0}+P', {Z_0}+Z')\rangle +\langle N' S_{2}({N_0}+N', {P_0}+P', {Z_0}+Z')\rangle \end{aligned}$$

17 $$\begin{aligned}{}&\frac{\partial }{\partial t}{\langle P'Z'\rangle }+(\langle u\rangle +k_{n})\frac{\partial {\langle P'Z'\rangle } }{\partial x}+(\langle v\rangle +k_{n})\frac{\partial {\langle P'Z'\rangle } }{\partial y}+(\langle w\rangle +w_{n})\frac{\partial {\langle P'Z'\rangle } }{\partial z}-{k_h}\frac{\partial ^2 {\langle P'Z'\rangle }}{\partial x^2}\nonumber \\&\quad -{k_h}\frac{\partial ^2 {\langle P'Z'\rangle }}{\partial y^2}-{k_v}\frac{\partial ^2 {\langle P'Z'\rangle }}{\partial z^2}=\langle P' S_{3}({N_0}+N', {P_0}+P', {Z_0}+Z')\rangle +\langle Z' S_{2}({N_0}+N', {P_0}+P', {Z_0}+Z')\rangle \end{aligned}$$

18 $$\begin{aligned}{}&\frac{\partial }{\partial t}{\langle N'Z'\rangle }+(\langle u\rangle +k_{n})\frac{\partial {\langle N'Z'\rangle } }{\partial x}+(\langle v\rangle +k_{n})\frac{\partial {\langle N'Z'\rangle } }{\partial y}+(\langle w\rangle +w_{n})\frac{\partial {\langle N'Z'\rangle } }{\partial z}-{k_h}\frac{\partial ^2 {\langle N'Z'\rangle }}{\partial x^2}\nonumber \\&\quad -{k_h}\frac{\partial ^2 {\langle N'Z'\rangle }}{\partial y^2}-{k_v}\frac{\partial ^2 {\langle N'Z'\rangle }}{\partial z^2} =\langle N' S_{3}({N_0}+N', {P_0}+P', {Z_0}+Z')\rangle +\langle Z' S_{1}({N_0}+N', {P_0}+P', {Z_0}+Z')\rangle . \end{aligned}$$

We have assumed that the random variables ${\textbf {N,\,P}}$ and ${\textbf {Z}}$ follow a joint lognormal probability distribution whose observed values are N, P, Z (densities of nutrient, phytoplankton and zooplankton respectively). The lognormal distribution can be fitted well to empirical data and has been widely used in continuous model^5^. We have ignored the third and higher order fluctuating terms to obtain simple closure.

Equations (10–12) represent the time evolution of mean terms, Eqs. (13–15) represent time evolution of variance terms and Eqs. (16–18) give the time evolution of covariance terms. Now the corresponding bio-physical model is

19 $$\begin{aligned}{}&\frac{d N_0}{dt}=\langle S_1 \rangle , \frac{d P_0}{dt}=\langle S_2 \rangle , \frac{d Z_0}{dt}=\langle S_3 \rangle ,\frac{d \langle N'^2 \rangle }{dt}=\langle N' S_1 \rangle , \frac{d \langle P'^2 \rangle }{dt}=\langle P' S_2 \rangle , \frac{d \langle Z'^2 \rangle }{dt}=\langle Z' S_3 \rangle ,\nonumber \\&\frac{d \langle N'P'\rangle }{dt}=\langle P'S_1 \rangle +\langle N'S_2 \rangle ,\nonumber \\&\frac{d \langle P'Z'\rangle }{dt}=\langle Z'S_2 \rangle +\langle P'S_3 \rangle ,\frac{d \langle N'Z'\rangle }{dt}=\langle Z'S_1 \rangle +\langle N'S_3 \rangle . \end{aligned}$$

Summing up first three equations of the above system provides

$$\begin{aligned} \frac{d N_0}{dt}+\frac{d P_0}{dt}+\frac{d Z_0}{dt}=0\Rightarrow {N_0}+{P_0}+{Z_0}=A\,(\text {constant}),\text {where}\, A\,\text {is total biomass of the system} \end{aligned}$$

since $\sum _{i=1}^{3} \langle S_{i}\rangle =\langle \sum _{i=1}^{3} S_{i} \rangle =0$. Adding next three and twice of last three equations of ODE system, we obtain

$$\begin{aligned} \langle N'^2 \rangle +\langle P'^2 \rangle +\langle Z'^2 \rangle +2\langle N'P'\rangle +2\langle P'Z'\rangle +2\langle N'Z'\rangle =B\,(\text {constant}), \end{aligned}$$

where *B* is the variance of the sum of all fluctuating components. Therefore, ${N_0}+{P_0}+{Z_0}$ and $\langle N'^2\rangle +\langle P'^2\rangle +\langle Z'^2\rangle +2 \langle N'P'\rangle +2 \langle P'Z'\rangle + 2\langle N'Z'\rangle$ are temporary conserved quantities. With appropriate scaling, the nine Eqs. (10–18) can be reduced to seven equations of dimensionless parameters and variables as

$$\begin{aligned} \frac{\partial p_0}{\partial T}=&-\frac{(\langle u\rangle +k_{p})}{V_m h}\frac{\partial p_0}{\partial X}-\frac{(\langle v\rangle +k_{p})}{V_m h}\frac{\partial p_0}{\partial Y}-\frac{(\langle w\rangle +w_p)}{V_m l}\frac{\partial p_0}{\partial Z}+\frac{k_{h}}{V_m h^{2}}\left( \frac{{\partial }^2 p_0}{\partial x^2}+\frac{{\partial }^2 p_0}{\partial y^2}\right) \\&+\frac{k_{v}}{V_m l^{2}}\left( \frac{{\partial }^2 p_0}{\partial z^2}\right) +{f_1}(V),\\ \frac{\partial z_0}{\partial T}=&-\frac{(\langle u\rangle +k_{z})}{V_m h}\frac{\partial z_0}{\partial X}-\frac{(\langle v\rangle +k_{z})}{V_m h}\frac{\partial z_0}{\partial Y}-\frac{(\langle w\rangle +w_z)}{V_m l}\frac{\partial z_0}{\partial Z}+\frac{k_{h}}{V_m h^{2}}\left( \frac{{\partial }^2 z_0}{\partial X^2}+\frac{{\partial }^2 z_0}{\partial Y^2}\right) \\&+\frac{k_{v}}{V_m l^{2}}\frac{{\partial }^2 Z_0}{\partial Z^2}+{f_2}(V),\\ \frac{\partial \langle p'^2 \rangle }{\partial T}=&-\frac{(\langle u\rangle +k_{p})}{V_m h}\frac{\partial \langle p'^2 \rangle }{\partial X}-\frac{(\langle v\rangle +k_{p})}{V_m h}\frac{\partial \langle p'^2 \rangle }{\partial Y}-\frac{(\langle w\rangle +w_p)}{V_m l}\frac{\partial \langle p'^2 \rangle }{\partial Z} +\frac{k_{v}}{V_m l^2}\frac{{\partial }^2 \langle p'^2 \rangle }{\partial Z^2}\\&+\frac{k_{h}}{V_m h^2}\left( \frac{{\partial }^2 \langle p'^2 \rangle }{\partial X^2}+\frac{{\partial }^2 \langle p'^2 \rangle }{\partial Y^2}\right) +{f_3}(V),\\ \frac{\partial \langle z'^2 \rangle }{\partial T}=&-\frac{(\langle u\rangle +k_{z})}{V_m h}\frac{\partial \langle z'^2 \rangle }{\partial X}-\frac{(\langle v\rangle +k_{z})}{V_m h}\frac{\partial \langle z'^2 \rangle }{\partial Y}-\frac{(\langle w\rangle +w_z)}{V_m l}\frac{\partial \langle z'^2\rangle }{\partial Z}\\&+\frac{k_{v}}{V_m l^2}\frac{{\partial }^2 \langle z'^2 \rangle }{\partial Z^2}+\frac{k_{h}}{V_m h^2}\left( \frac{{\partial }^2 \langle z'^2 \rangle }{\partial X^2}+\frac{{\partial }^2 \langle z'^2 \rangle }{\partial Y^2}\right) +{f_4}(V),\\ \frac{\partial \langle n'^2\rangle }{\partial T}=&-\frac{(\langle u\rangle +k_{N})}{V_m h}\frac{\partial \langle n'^2\rangle }{\partial X}-\frac{(\langle v\rangle +k_{N})}{V_m h}\frac{\partial \langle n'^2\rangle }{\partial Y}-\frac{(\langle w\rangle +w_N)}{V_m l}\frac{\partial \langle n'^2 \rangle }{\partial Z}+\frac{k_{v}}{V_m l^2}\frac{{\partial }^2 \langle n'^2\rangle }{\partial Z^2}\\&+\frac{k_{h}}{V_m h^2}\left( \frac{{\partial }^2 \langle n'^2 \rangle }{\partial X^2}+\frac{{\partial }^2 \langle n'^2\rangle }{\partial Y^2}\right) +{f_5}(V),\\ \frac{\partial \langle p'z'\rangle }{\partial T}=&-\frac{(\langle u\rangle +k_{pz})}{V_m h}\frac{\partial \langle p'z'\rangle }{\partial X}-\frac{(\langle v\rangle +k_{pz})}{V_m h}\frac{\partial \langle p'z'\rangle }{\partial Y}-\frac{(\langle w\rangle +w_{pz})}{V_m l}\frac{\partial \langle p'z'\rangle }{\partial Z}\\&+\frac{k_{v}}{V_m l^2 }\frac{{\partial }^2 \langle p'z'\rangle }{\partial Z^2}+\frac{k_{h}}{V_m h^2}\left( \frac{{\partial }^2 \langle p'z'\rangle }{\partial X^2}+\frac{{\partial }^2 \langle p'z'\rangle }{\partial Y^2}\right) +{f_6}(V),\\ \frac{\partial \langle n'p'\rangle }{\partial T}=&-\frac{(\langle u\rangle +k_{np})}{V_m h}\frac{\partial \langle n'p'\rangle }{\partial X}-\frac{(\langle v\rangle +k_{np})}{V_m h}\frac{\partial \langle n'p'\rangle }{\partial Y}-\frac{(\langle w\rangle +w_{np})}{V_m l}\frac{\partial \langle n'p'\rangle }{\partial Z} +\frac{k_{v}}{V_m l^2}\frac{{\partial }^2 \langle n'p'\rangle }{\partial Z^2}\\&+\frac{k_{h}}{V_m h^2}\left( \frac{{\partial }^2 \langle n'p'\rangle }{\partial X^2}+\frac{{\partial }^2 \langle n'p'\rangle }{\partial Y^2}\right) +{f_7}(V), \end{aligned}$$

where $V=(p_0, n_0, \langle p'^2\rangle , \langle p'^2\rangle , \langle z'^2\rangle , \langle n'^2\rangle , \langle p'z'\rangle , \langle n'p'\rangle )$ and $f_1, f_2,..,f_7$ are functional forms of *V* provided in supplementary information (SI). All dimensionless parameters and variables are defined in Table 2. Clearly,

$$\begin{aligned} {p_0}+{n_0}+{z_0}=1,\,\,\langle p'^2 \rangle +\langle n'^2 \rangle +\langle z'^2 \rangle +2\langle p'z'\rangle +2\langle n'z'\rangle +2\langle n'p'\rangle =1. \end{aligned}$$

### Model analysis

Let, ${V^*}=({p_0}^{*},{z_0}^{*},{\langle {p'^{2}}\rangle }^{*},{\langle z'^{2}\rangle }^{*},{\langle n'^{2} \rangle }^{*},{\langle p'z' \rangle }^{*}, {\langle n'p' \rangle }^{*})$ be a spatially homogeneous steady state of dimensionless reaction-advection-diffusion closure system. Then,

$$\begin{aligned}{}&\text {(I)}\,\frac{\partial {p_0}^{*}}{\partial t}=0, \frac{\partial {p_0}^{*}}{\partial x}=0,\frac{\partial {p_0}^{*}}{\partial y}=0, \frac{\partial {p_0}^{*}}{\partial z}=0,\frac{\partial {z_0}^{*}}{\partial t}=0, \frac{\partial {z_0}^{*}}{\partial x}=0, \frac{\partial {z_0}^{*}}{\partial y}=0,\frac{\partial {z_0}^{*}}{\partial z}=0;\\&\text {(II)}\,\frac{\partial {\langle {p'^{2}}\rangle }^{*}}{\partial t}=0, \frac{\partial {\langle {p'^{2}}\rangle }^{*}}{\partial x}=0, \frac{\partial {\langle {p'^{2}}\rangle }^{*}}{\partial y}=0, \frac{\partial {\langle {p'^{2}}\rangle }^{*}}{\partial z}=0;\\&\quad \frac{\partial {\langle {z'^{2}}\rangle }^{*}}{\partial t}=0, \frac{\partial {\langle {z'^{2}}\rangle }^{*}}{\partial x}=0,\frac{\partial {\langle {z'^{2}}\rangle }^{*}}{\partial y}=0, \frac{\partial {\langle {z'^{2}}\rangle }^{*}}{\partial z}=0;\\&\quad \frac{\partial {\langle {n'^{2}}\rangle }^{*}}{\partial t}=0, \frac{\partial {\langle {n'^{2}}\rangle }^{*}}{\partial x}=0,\frac{\partial {\langle {n'^{2}}\rangle }^{*}}{\partial y}=0, \frac{\partial {\langle {n'^{2}}\rangle }^{*}}{\partial z}=0;\\&\text {(III)}\,\frac{\partial {\langle n'p'\rangle }^{*}}{\partial t}=0, \frac{\partial {\langle n'p'\rangle }^{*}}{\partial x}=0, \frac{\partial {\langle n'p'\rangle }^{*}}{\partial y}=0, \frac{\partial {\langle n'p'\rangle }^{*}}{\partial z}=0;\\&\quad \frac{\partial {\langle p'z'\rangle }^{*}}{\partial t}=0, \frac{\partial {\langle p'z'\rangle }^{*}}{\partial x}=0,\frac{\partial {\langle p'z'\rangle }^{*}}{\partial y}=0,\frac{\partial {\langle p'z'\rangle }^{*}}{\partial z}=0. \end{aligned}$$

We define,

$$\begin{aligned}{}&Q_1(x,y,z,t)={p_0}(x,y,z,t)-{p_0}^{*},\,Q_2(x,y,z,t)={z_0}(x,y,z,t)-{z_0}^{*},\\&Q_3(x,y,z,t)=\langle p'^2\rangle (x,y,z,t)-{\langle {p'^2}\rangle }^{*},\\&Q_4(x,y,z,t)=\langle z'^2\rangle (x,y,z,t)-{\langle z'^2\rangle }^{*},\\&Q_5(x,y,z,t)=\langle n'^2\rangle (x,y,z,t)-{\langle n'^2\rangle }^{*},\,Q_6(x,y,z,t)=\langle p'z'\rangle (x,y,z,t)-{\langle p'z'\rangle }^{*},\\&Q_7(x,y,z,t)=\langle n'p'\rangle (x,y,z,t)-{\langle n'p'\rangle }^{*} \end{aligned}$$

to be a small inhomogeneous perturbations about the steady state $V^*$. Provided these perturbations are sufficiently small, it is again possible to linearize the dimensionless reaction-advection-diffusion closure system about the homogeneous steady state $V^*$. Now the Jacobian of the linearized system about the steady state is

$$\begin{aligned} J =\left( {a_{ij}}\right) _{7\times 7}= \begin{pmatrix} \frac{\partial f_1}{\partial p_0}&{}\frac{\partial f_1}{\partial z_0}&{}\frac{\partial f_1}{\partial \langle p'2\rangle }&{}\frac{\partial f_1}{\partial \langle z'^2\rangle }&{}\frac{\partial f_1}{\partial \langle n'^2\rangle }&{}\frac{\partial f_1}{\partial \langle n'p'\rangle }&{}\frac{\partial f_1}{\partial \langle p'z'\rangle }\\ \\ \frac{\partial f_2}{\partial p_0}&{}\frac{\partial f_2}{\partial z_0}&{}\frac{\partial f_2}{\partial \langle p'2\rangle }&{}\frac{\partial f_2}{\partial \langle z'^2\rangle }&{}\frac{\partial f_2}{\partial \langle n'^2\rangle }&{}\frac{\partial f_2}{\partial \langle n'p'\rangle }&{}\frac{\partial f_2}{\partial \langle p'z'\rangle }\\ \\ \frac{\partial f_3}{\partial p_0}&{}\frac{\partial f_3}{\partial z_0}&{}\frac{\partial f_3}{\partial \langle p'2\rangle }&{}\frac{\partial f_3}{\partial \langle z'^2\rangle }&{}\frac{\partial f_3}{\partial \langle n'^2\rangle }&{}\frac{\partial f_3}{\partial \langle n'p'\rangle }&{}\frac{\partial f_3}{\partial \langle p'z'\rangle }\\ \\ \frac{\partial f_4}{\partial p_0}&{}\frac{\partial f_4}{\partial z_0}&{}\frac{\partial f_4}{\partial \langle p'2\rangle }&{}\frac{\partial f_4}{\partial \langle z'^2\rangle }&{}\frac{\partial f_4}{\partial \langle n'^2\rangle }&{}\frac{\partial f_4}{\partial \langle n'p'\rangle }&{}\frac{\partial f_4}{\partial \langle p'z'\rangle }\\ \\ \frac{\partial f_5}{\partial p_0}&{}\frac{\partial f_5}{\partial z_0}&{}\frac{\partial f_5}{\partial \langle p'2\rangle }&{}\frac{\partial f_5}{\partial \langle z'^2\rangle }&{}\frac{\partial f_5}{\partial \langle n'^2\rangle }&{}\frac{\partial f_5}{\partial \langle n'p'\rangle }&{}\frac{\partial f_5}{\partial \langle p'z'\rangle }\\ \\ \frac{\partial f_6}{\partial p_0}&{}\frac{\partial f_6}{\partial z_0}&{}\frac{\partial f_6}{\partial \langle p'2\rangle }&{}\frac{\partial f_6}{\partial \langle z'^2\rangle }&{}\frac{\partial f_6}{\partial \langle n'^2\rangle }&{}\frac{\partial f_6}{\partial \langle n'p'\rangle }&{}\frac{\partial f_6}{\partial \langle p'z'\rangle }\\ \\ \frac{\partial f_7}{\partial p_0}&{}\frac{\partial f_7}{\partial z_0}&{}\frac{\partial f_7}{\partial \langle p'2\rangle }&{}\frac{\partial f_7}{\partial \langle z'^2\rangle }&{}\frac{\partial f_7}{\partial \langle n'^2\rangle }&{}\frac{\partial f_7}{\partial \langle n'p'\rangle }&{}\frac{\partial f_7}{\partial \langle p'z'\rangle }\\ \end{pmatrix}_{V^*}. \end{aligned}$$

Hence, the linearized equations of the closure system can be written as

$$\begin{aligned} \frac{\partial Q_1 }{\partial T}=&\sum _{i=1}^{7} {a_{1i}} Q_i-\frac{\left( \langle u\rangle +{k_{p}}\right) }{V_m h}\frac{\partial Q_l }{\partial X}-\frac{\left( \langle v\rangle +{k_{p}}\right) }{V_m h}\frac{\partial Q_l }{\partial Y} -\frac{\left( \langle w\rangle +{w_{p}}\right) }{V_m l}\frac{\partial Q_l }{\partial Z} \\&+\frac{k_h}{V_m h^2}\left( \frac{\partial ^2 Q_l}{\partial X^2}+\frac{\partial ^2 Q_l}{\partial Y^2}\right) +\frac{k_v}{V_m l^2}\left( \frac{\partial ^2 Q_l }{\partial Z^2}\right) ,\\ \frac{\partial Q_2 }{\partial T}=&\sum _{i=1}^{7} {a_{2i}} Q_i-\frac{\left( \langle u\rangle +{k_{z}}\right) }{V_m h}\frac{\partial Q_2 }{\partial X}-\frac{\left( \langle v\rangle +{k_{p}}\right) }{V_m h}\frac{\partial Q_l }{\partial Y} -\frac{\left( \langle w\rangle +{w_{z}}\right) }{V_m l}\frac{\partial Q_2 }{\partial Z} \\&+\frac{k_h}{V_m h^2}\left( \frac{\partial ^2 Q_2}{\partial X^2}+\frac{\partial ^2 Q_2}{\partial Y^2}\right) +\frac{k_v}{V_m l^2}\left( \frac{\partial ^2 Q_2 }{\partial Z^2}\right) ,\\ \frac{\partial Q_3 }{\partial T}=&\sum _{i=1}^{7} {a_{3i}} Q_i-\frac{\left( \langle u\rangle +{k_{p}}\right) }{V_m h}\frac{\partial Q_3 }{\partial X}-\frac{\left( \langle v\rangle +{k_{p}}\right) }{V_m h}\frac{\partial Q_3 }{\partial Y} -\frac{\left( \langle w\rangle +{w_{p}}\right) }{V_m l}\frac{\partial Q_3 }{\partial Z} \\&+\frac{k_h}{V_m h^2}\left( \frac{\partial ^2 Q_3}{\partial X^2}+\frac{\partial ^2 Q_3}{\partial Y^2}\right) +\frac{k_v}{V_m l^2}\left( \frac{\partial ^2 Q_3 }{\partial Z^2}\right) , \end{aligned}$$

$$\begin{aligned} \frac{\partial Q_4 }{\partial t}=&\sum _{i=1}^{7} {a_{4i}} Q_i-\frac{\left( \langle u\rangle +{k_{z}}\right) }{V_m h}\frac{\partial Q_4 }{\partial X}-\frac{\left( \langle v\rangle +{k_{z}}\right) }{V_m h}\frac{\partial Q_4 }{\partial Y} -\frac{\left( \langle w\rangle +{w_{z}}\right) }{v_m l}\frac{\partial Q_4 }{\partial Z} +\frac{k_h}{V_m h^2}\left( \frac{\partial ^2 Q_4}{\partial X^2}+\frac{\partial ^2 Q_4}{\partial Y^2}\right) \\&+\frac{k_v}{V_m l^2}\left( \frac{\partial ^2 Q_4 }{\partial Z^2}\right) ,\\ \frac{\partial Q_5 }{\partial T}=&\sum _{i=1}^{7} {a_{5i}} Q_i-\left( \langle u\rangle +{k_{n}}\right) \frac{\partial Q_5 }{\partial X}-\left( \langle v\rangle +{k_{n}}\right) \frac{\partial Q_5}{\partial Y} -\left( \langle w\rangle +{w_{n}}\right) \frac{\partial Q_5}{\partial Z} +{k_h}\left( \frac{\partial ^2 Q_5}{\partial X^2}+\frac{\partial ^2 Q_5}{\partial Y^2}\right) +{k_v}\left( \frac{\partial ^2 Q_5 }{\partial Z^2}\right) ,\\ \frac{\partial Q_6 }{\partial T}=&\sum _{i=1}^{7} {a_{6i}} Q_i-\frac{\left( \langle u\rangle +{k_{pz}}\right) }{v_m h}\frac{\partial Q_6 }{\partial X}-\frac{\left( \langle v\rangle +{k_{pz}}\right) }{V_m h}\frac{\partial Q_6 }{\partial Y} -\frac{\left( \langle w\rangle +{w_{pz}}\right) }{V_m l}\frac{\partial Q_6}{\partial Z}\\&+\frac{k_h}{V_m h^2}\left( \frac{\partial ^2 Q_6}{\partial X^2}+\frac{\partial ^2 Q_6}{\partial Y^2}\right) +\frac{k_v}{V_m l^2}\left( \frac{\partial ^2 Q_6}{\partial Z^2}\right) ,\\ \frac{\partial Q_7 }{\partial T}=&\sum _{i=1}^{7} {a_{7i}} Q_i-\frac{\left( \langle u\rangle +{k_{np}}\right) }{V_m h}\frac{\partial Q_7}{\partial X}-\frac{\left( \langle v\rangle +{k_{np}}\right) }{V_m h}\frac{\partial Q_7}{\partial Y} -\frac{\left( \langle w\rangle +{w_{np}}\right) }{V_m l}\frac{\partial Q_7}{\partial Z} \\&+\frac{k_h}{V_m h^2}\left( \frac{\partial ^2 Q_7}{\partial X^2}+\frac{\partial ^2 Q_7}{\partial Y^2}\right) +\frac{k_v}{V_m l^2}\left( \frac{\partial ^2 Q_7}{\partial Z^2}\right) . \end{aligned}$$

We now substitute, $Q_i={\alpha _i}\,e^{-\lambda t}e^{-\sigma _1 x} e^{-\sigma _{2} y} e^{-\sigma _{3} z}$, $\lambda ,{\sigma _1},{\sigma _2},{\sigma _3}>0$ in the above system and obtain 7 equations in $\alpha _i$, which can be written in matrix form as

$$\begin{aligned} S\times \alpha =\textbf{0}, \end{aligned}$$

where $\textbf{0}$ is zero vector of order 7, $\alpha =\left( \alpha _1,...,\alpha _7\right) ^{\text {T}}$ is a column vector and *S* is matrix of order $7\times 7$, where all non-diagonal elements are same for the matrices *S* and the Jacobian (*J*) of the linearized closure system about the point ${V^*}$. The diagonal elements $s_{ii}$ are as follows:

$$\begin{aligned} s_{11}=&\lambda +a_{11}+\frac{\langle u\rangle +{k_p}}{V_m h} {\sigma _1}+\frac{\langle v\rangle +{k_p}}{V_m h} {\sigma _2}+\frac{\langle w\rangle +{w_p}}{V_m l} {\sigma _3}+\frac{k_h}{V_m h^2}({\sigma _1}^{2}+{\sigma _2}^{2})+\frac{k_v}{V_m l^2} {\sigma _3}^{2} ;\\ s_{22}=&\lambda +a_{22}+\frac{\langle u\rangle +{k_z}}{V_m h} {\sigma _1}+\frac{\langle v\rangle +{k_z}}{V_m h} {\sigma _2}+\frac{\langle w\rangle +{w_z}}{V_m l} {\sigma _3}+\frac{k_h}{V_m h^2}({\sigma _1}^{2}+{\sigma _2}^{2})+\frac{k_v}{V_m l^2} {\sigma _3}^{2};\\ s_{33}=&\lambda +a_{33}+\frac{\langle u\rangle +{k_p}}{V_m h} {\sigma _1}+\frac{\langle v\rangle +{k_p}}{V_m h} {\sigma _2}+\frac{\langle w\rangle +{w_p}}{V_m l} {\sigma _3}+\frac{k_h}{V_m h^2}({\sigma _1}^{2}+{\sigma _2}^{2})+\frac{k_v}{V_m l^2} {\sigma _3}^{2};\\ s_{44}=&\lambda +a_{44}+\frac{\langle u\rangle +{k_z}}{V_m h} {\sigma _1}+\frac{\langle v\rangle +{k_z}}{V_m h} {\sigma _2}+\frac{\langle w\rangle +{w_z}}{V_m l} {\sigma _3}+\frac{k_h}{V_m h^2}({\sigma _1}^{2}+{\sigma _2}^{2})+\frac{k_v}{V_m l^2} {\sigma _3}^{2};\\ s_{55}=&\lambda +a_{55}+\frac{\langle u\rangle +{k_n}}{V_m h} {\sigma _1}+\frac{\langle v\rangle +{k_n}}{V_m h} {\sigma _2}+\frac{\langle w\rangle +{w_n}}{V_m l} {\sigma _3}+\frac{k_h}{V_m h^2}({\sigma _1}^{2}+{\sigma _2}^{2})+\frac{k_v}{V_m l^2} {\sigma _3}^{2};\\ s_{66}=&\lambda +a_{11}+\frac{\langle u\rangle +{k_pz}}{V_m h} {\sigma _1}+\frac{\langle v\rangle +{k_pz}}{V_m h} {\sigma _2}+\frac{\langle w\rangle +{w_pz}}{V_m l} {\sigma _3}+\frac{k_h}{V_m h^2}({\sigma _1}^{2}+{\sigma _2}^{2})+\frac{k_v}{V_m l^2} {\sigma _3}^{2};\\ s_{77}=&\lambda +a_{11}+\frac{\langle u\rangle +{k_np}}{V_m h} {\sigma _1}+\frac{\langle v\rangle +{k_np}}{V_m h} {\sigma _2}+\frac{\langle w\rangle +{w_np}}{V_m l} {\sigma _3}+\frac{k_h}{V_m h^2}({\sigma _1}^{2}+{\sigma _2}^{2})+\frac{k_v}{V_m l^2} {\sigma _3}^{2}. \end{aligned}$$

We now obtain the characteristic equation of the linearized closure system as $\text {Det}(S)=0$ for some suitable choices of $\sigma _1, \sigma _2, \sigma _3$ and $\alpha \ne \textbf{0}$.

#### Analysis of critical threshold value

We now consider a particular set of parameter values (to be estimated) representing a particular circumstantial scenario and a suitable set for amplitudes $\sigma _1, \sigma _2, \sigma _3$. For aquatic ecosystem, $k_{p}, w_{p}$ are swimming and sinking velocities of phytoplankton community, which only depend on the nature of existing or dominating phytoplankton species, hence they do not vary with seasonal or environmental factors. Therefore, among all advection and diffusion factors, $k_h, k_v, \langle u\rangle , \langle v\rangle , \langle w\rangle$ are the control parameters. Hence, depending on the estimated parameter set, we consider the dominating control parameter to obtain its corresponding critical value.

Let us assume that, for a considered set, $k_v, \langle w\rangle$ are the dominating control parameters of the system. Keeping $k_v$ as a variable and putting other values, we determine *S*. Thus, $\text {Det} (S)=0$ provides the characteristic equation

$$\begin{aligned} f[k_v](\lambda )=\sum _{k=0}^{7} {a_k}{\lambda }^k=0, \end{aligned}$$

of degree 7, where each coefficient is a polynomial of $k_h$. Here,

$$\begin{aligned} {a_0}=1, {a_k}=\sum _{j=0}^{k}{b_j}{k_h}^j,k=1,2,...,7. \end{aligned}$$

Our aim is to construct a Routh Hurwitz matrix from this polynomial. Degree of the characteristic polynomial is 7, therefore dimension of the Routh-Hurwitz matrix will be $7\times 4$. Elements of first two rows are coefficient of the characteristic polynomial, other elements are determined by cross multiplying the coefficients of previous rows.

We now proceed with Routh Hurwitz stability criteria to find a domain of $k_v$, which will cause the spatially uniform steady state to be stable. The elements of Routh-Hurwitz matrix are polynomial functions of $k_v$ and nature of stability of spatially uniform steady state along with the system depends on the nature of eigenvalues, which gets decided by the sign of elements of first column of Routh Hurwitz matrix. This provides a lower bound on the domain of $k_v$, say, ${k_v}^c$. Whenever ${k_v}>{k_v}^c$, eigenvalues are real and negative or they have negative real parts, hence spatially uniform steady state $V^{*}$ becomes stable for ${k_v}>{k_v}^c$ and whenever this condition gets violated, $V^{*}$ becomes unstable. Whenever $V^{*}$ is stable, corresponding dynamics of phytoplankton biomass distribution remains consistent. Now, whatever the nature of distribution is, whenever it is consistent under the fact $V^{*}$ is stable for ${k_v}>{k_v}^c$, the corresponding time series graph of phytoplankton biomass (mean $p_0$) decides the nature of phytoplankton bloom development. When we choose ${k_v}<{k_v}^c$, stability criteria of considered spatially homogeneous steady state is violated, hence whatever the dynamics of phytoplankton was corresponding to $V^{*}$, is also violated but in this zone, where stability criteria gets violated for $V^{*}$, there occurs one more spatially homogenous steady state, say, ${V_1}^{*}$, whose domain of stability depending on $k_v$ intersects with this unstable domain corresponding to ${k_v}<{k_v}^c$ for $V^{*}$. If we follow the same stability analysis around ${V_1}^{*}$, we will be able to observe this and we continue in this manner till the critical parameter values corresponding to dominating factors become negative. Therefore, whenever $V^*$ is unstable in a domain, we get another spatially uniform steady state whose stable domain intersects with unstable domain of $V^*$ and stability of ${V_1}^{*}$ in the unstable domain of $V^{*}$ causes changes in dynamical distribution of phytoplankton biomass along with other mean and variances. This provides a different dynamics for phytoplankton biomass, in fact, every time stability domain breaks for each ${V_i}^{*}$, we will get a new stable domain intersecting the unstable domain of ${V_{(i-1)}}^{*}$ and corresponding dynamics of distribution of variables will depend on the nature of spatially uniform steady state, say ${V_i}^{*}$, at that moment. This even stands for variations in phytoplankton distribution at different water layers. Hence, for each case, the relation between the critical value ${k_v}^c$ and existing value of dominating quantity $k_v$ determines the nature of interaction in the NPZ aquatic ecosystem. We now choose a value for $k_v$ higher than ${k_v}^c$ and treating $\langle w\rangle$ as a variable, we get the critical value $w_c$ for $\langle w\rangle$. These critical values are provided in (SI Tables 1, 2).

## Boundary and initial conditions

To conserve the property of closure model, zero flux boundary condition has been introduced to the system. While non-dimensionalizing the system we have considered *h* is the length scale along x, y directions and *l* is the depth scale along z direction, in particular, we have considered a particular cubic part of an aquatic system having volume $h^2{l}$ $\text {meter}^3$.

According to zero-flux boundary condition, rate of change of model variables along vertical and horizontal directions remains zero at boundary points implying

$$\begin{aligned}{}&\frac{\partial {p_0}}{\partial X}|_{(0,0,0,T)}=0,\frac{\partial {p_0}}{\partial Y}|_{(0,0,0,T)}=0,\frac{\partial {p_0}}{\partial Z}|_{(0,0,0,T)}=0, \frac{\partial {p_0}}{\partial X}|_{(h,0,0,T)}=0,\frac{\partial {p_0}}{\partial Y}|_{(0,h,0,T)}=0,\frac{\partial {p_0}}{\partial Z}|_{(0,0,l,T)}=0. \end{aligned}$$

The remaining variables $z_0, {\langle p'^2 \rangle },{\langle z'^2 \rangle },{\langle n'^2 \rangle },{\langle n'p' \rangle },{\langle p'z' \rangle }$ satisfy the same conditions. The initial conditions are

$$\begin{aligned}{}&{p_0}(X,Y,Z, 0)={\phi _1}(X, Y, Z)={C_1}e^{-\frac{Z^2}{l^{\gamma }}},\,\,{z_0}(X, Y, Z,0)={C_2}e^{-\frac{Z^2}{l^{\gamma }}},\\&{\langle p'^2 \rangle }(X, Y, Z, 0)={\phi _3}(X, Y, Z)={C_3}e^{-\frac{Z^2}{l^{\gamma }}}e^{-\frac{X^2+Y^2}{h^2}},\,\, {\langle z'^2 \rangle }(X, Y, Z, 0)={\phi _4}(x,y,z)={C_4}e^{-\frac{Z^2}{l^{\gamma }}}e^{-\frac{X^2+Y^2}{h^2}},\\&{\langle n'^2 \rangle }(X, Y, Z, 0)={\phi _5}(x,y,z)={C_5}e^{-\frac{Z^2}{l^{\gamma }}}e^{-\frac{X^2+Y^2}{h^2}},\,\, {\langle n'p' \rangle }(X, Y, Z, 0)={C_6},\,\, {\langle p'z' \rangle }(X, Y, Z, 0)={C_7},\\&\text {where}\,\gamma =\frac{\sigma _3}{2}\,\text { and }C_i,\,i=1,...,7\,\text {are non-negative constants}. \end{aligned}$$

In the formulated model variables, all mean and variance terms are always non-negative, whereas covariance terms can take any sign (depending on the relation between the corresponding model variables) and, corresponding dimensionless variables also follow the same. These dimensionless terms satisfy the condition, $\langle p'^2 \rangle +\langle n'^2\rangle +\langle z'^2\rangle +2\langle p'z'\rangle +2\langle n'z'\rangle +2\langle n'p'\rangle =1$. Hence, distribution of these variables are bounded by 1. On the other hand, phytoplankton mean decreases with depth, depending on that, mean of herbivorous zooplankton varies, both are bounded by constant 1 (${p_0}+{n_0}+{z_0}=1$) for dimensionless system and all these dimensionless quantities $p_0, n_0, z_0$ are strictly non-negative. Therefore, the functions $\phi _1,...,\phi _7$ are chosen in such a way so that they satisfy these criteria.

Besides, in this model it is assumed that only phytoplankton community has swimming and sinking velocities, hence $k_{p}, w_{p}>0$, whereas $k_{n}=0, w_{n}=0, k_{z}=0, w_{z}=0$. Also, we have assumed that $k_{np}=0, w_{np}=0$, $k_{pz}=0, w_{pz}=0$ (since we lack any observational or theoretical basis for modelling the effect of swimming and sinking on covariance).

## Critical depth ($D_c$)

The phytoplankton species existing in higher depth should have access to essential components like solar irradiance for the continuation of photosynthesis, and the availability of such ingredients depends on the value of the depth (say, $H_c$) at which sinking phytoplankton species get stuck because of the resilience of water body. For any water column, there exist a layer (say, $D_{c}$) beyond which total loss of phytoplankton biomass neutralizes the total growth of phytoplankton, so bloom cannot occur underneath this depth $D_c$. This depth is called critical depth. Depending on the value of this depth, the possibility of bloom occurrence in higher depth is decided. Also, depending on the fact whether $H_c$ is less than $D_c$ or not, the possibilities of the occurrence of bloom in higher depth within the water column vary. Critical depth ($D_c$) is calculated using a reformation of the Sverdrup equation^22^, namely,

20 $$\begin{aligned}{}&{D_c}=\frac{\sum {E_0}}{3.78 \kappa }, \end{aligned}$$

where $\sum {E_0}$ is the surface photosynthetically active radiation (PAR) integrated over 24h expressed in $\text {mol photons}\,m^{-2}\,{day^{-1}}$ and $\kappa$ is light attenuation coefficient.

**Table 2:** Dimension and ranges of different quantities used in this model and their dimensions.

Quantity	Dimension	Parameter	Dimensionless
		Values	Parameter
A	$\mu g N l^{-1}$	1^8^	–
$V_m$	$day^{-1}$	1–2.5^23^	–
K	$\mu g N l^{-1}$	0.6–1.8^24^	k $\left( =K/A\right)$
$m_p$	$day^{-1}$	0–0.15^25^	$\frac{m_p}{V_m}$
$I_0$	$W\,m^{-2}$	160–320^26^	–
$\kappa$	$m^{-1}$	0.005–0.15^25^	–
r	$day^{-1}$	0.05–0.15^27^	$r/{V_m}$
s	$day^{-1}$	0.032–0.08^27^	$s/{V_m}$
$k_2$	$day^{-1}$	0.0008–0.13^27^	${k_2}/{V_m}$
$\tau _z$	$day^{-1}$	0.050112^28^	${\tau _z}/{V_m}$
$a_z$	–	0.2–0.5^27^	-
$g_z$	${day^{-1}}\,(\mu g N l^{-1})^{-1}$	0.4–1.4^24^	$\frac{g_z A}{V_m}$
$k_h$	$m^2\,day^{-1}$	0.01–0.2^21^	${k_h}/({V_m}*{h^2})$
$k_v$	$m^2\,day^{-1}$	0.001–10^21^	–
$\langle u\rangle$	$m\,day^{-1}$	–	–
$\langle v\rangle$	$m\,day^{-1}$	–	–
$\langle w\rangle$	$m\,day^{-1}$	–	–
$k_p$	$m\,day^{-1}$	0.01-5^29^	–
$w_p$	$m\,day^{-1}$	0.012-6^30^	–
$m_z$	${day^{-1}}\,(\mu g N l^{-1})^{-1}$	0-0.3^27^	${m_z} A/{V_m}$
$\alpha$	$\left( W\,{m^{-2}}\,{day^{-1}}\right) ^{-1}$	0.015^31^	–
*x*	*meter*	–	$X\,(=x/h)$
*y*	*meter*	–	$Y\,(=y/h)$
*z*	*meter*	–	$Z\,(=z/l)$
*t*	*day*	–	*T*

## Results

To capture the role of physical mixing process and spatial average of water velocity on bloom dynamics in global scale, our numerical investigation (the assigned parameter values are obtained from Table2) is sectioned into two parts,

(i) System 1: spatial average of water velocity is zero ($\langle u\rangle =\langle v\rangle =\langle w\rangle =0$), but turbulent diffusion is non-zero (${k_h}\ne 0,{k_v}\ne 0$), which is possible when mixing of comparatively highly dense or less dense external fluid or temperature difference causing a difference in water density which gives rise to diffusivity without influencing water velocity, like the lake, pond and almost calm marine ecosystem;
(ii) System 2: spatial average of water velocity along with turbulent diffusion is non-zero ($\langle u\rangle \ne 0,\,\langle v\rangle \ne 0,\,\langle w\rangle \ne 0,{k_h}\ne 0,{k_v}\ne 0$), which stands for a complete turbulent medium like restless marine environment and flowing river.

### Surface bloom (SB)

#### SB1: When phytoplankton community is buoyant in nature ($k_p>w_p$) and spatial average of water velocity is zero ($\langle u\rangle =\langle v\rangle =\langle w\rangle =0$)

**Figure 1:**
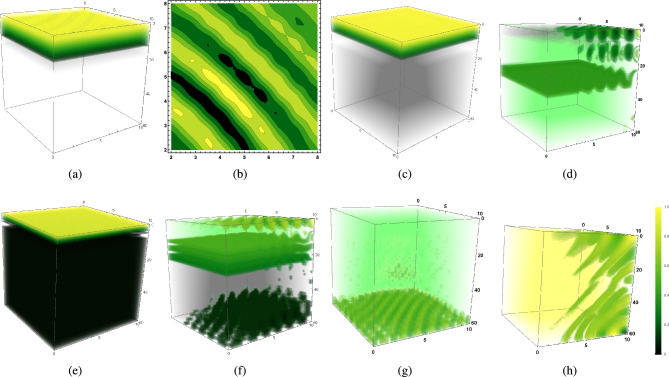
The figure shows the distribution of phytoplankton biomass ($p_0$) under different circumstances. (**a**) Vertical distribution of $p_0$ of buoyant community of system 1 ($k_v<{k_v}^c$), (**b**) Horizontal distribution of $p_0$ of the buoyant community of system 1 ($k_v<{k_v}^c$), (**c**) Vertical distribution of $p_0$ of system 1 ($k_v<{k_v}^c$), (**d**) Vertical distribution of $p_0$ of buoyant community of system 2 ($k_v<k_h, k_h<{k_h}^c, \langle u\rangle<u_c, \langle v\rangle <v_c$), (**e**) Vertical distribution of $p_0$ of buoyant community of system 2 ($k_v < k_h, k_h> {k_h}^c, \langle u\rangle> u_c, \langle v\rangle > v_c$, (**f**) Vertical distribution of $p_0$ of heavier community of system 2 ($k_v<k_h, k_h>{k_h}^c,$ $\langle u\rangle> u_c, \langle v\rangle >v_c$, (**g**) Vertical distribution of $p_0$ of heavier community of system 2 ($k_v>{k_v}^c, \langle w\rangle >w_c$) (**h**) Vertical distribution of $p_0$ of buoyant community of system 2 ($k_v>{k_v}^c, \langle w\rangle >w_c$). All the figures are in the presence of low grazing influence. The parameters values are mentioned in SI.

Figure 1a represents the vertical distribution of phytoplankton biomass of buoyant community ($k_p>w_p$) on the 60th day of the warmer season ($I_0=320\,W/m^2$) through a water column having depth $L_z=60$ meters in an aquatic medium of system 1. In contrast, vertical turbulent diffusion ($k_v$) is low as high surface irradiance $I_0=320\,W/m^2$ causes high thermal stratification of surface water resulting in reduced vertical eddy diffusivity. However, it dominates horizontal eddy diffusivity ($k_v>k_h$) in the absence of surface wind. Under such circumstances, the surface bloom of the buoyant community has been observed on the 60th day of warmer season while existing low vertical turbulence is highly dominated by its corresponding critical value, that is, $k_v<{k_v}^c$. Figure 1b represents the horizontal distribution of phytoplankton during that period, where we observe several yellow to dark green patches floating on surface water, which indicates intermittent patches of phytoplankton bloom covering the surface water. Here, the numerical results show that high buoyancy, in the presence of low grazing influence, exerts a positive impact on phytoplankton productivity and growth rate $V_m$, by driving dominating phytoplankton community to light and nutrient-rich zone (Fig. 1a,b).

#### SB2: When phytoplankton community has higher sinking tendency ($k_p<w_p$) and spatial average of water velocity is zero

**Figure 2:**
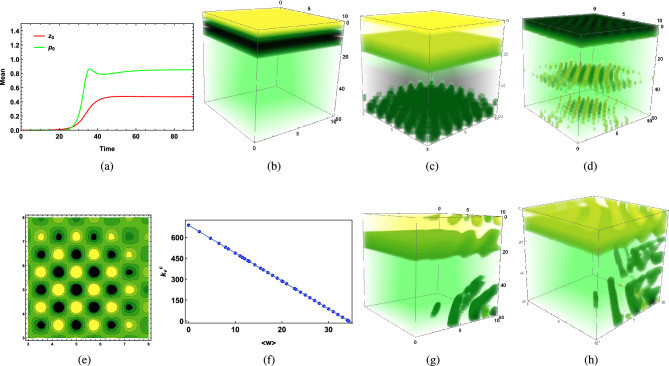
(**a**) Time variation of $p_0$ and $z_0$ in summer season of system 2, $p_0$ is buoyant in nature ($k_p>w_p$), ${k_h}>{k_h}^c, \langle u\rangle >{u_c},$ $\langle v\rangle >{v_c}$, (**b**) Vertical distribution of $z_0$ on 115th day of summer season of system 1, $p_0$ is heavier ($w_p>k_p$), ($k_v<{k_v}^c$), (**c**) Vertical distribution of $p_0$ on 60th day of cold season (${k_v}<{k_v}^c,<w> <{w_c}$) of system 2, $p_0$ has higher sinking tendency ($w_p>k_p$), (**d**) Vertical distribution of $p_0$ on 48th day of cold season of system 1 ($k_v<<{k_v}^c$), $p_0$ has higher sinking tendency, (**e**) Horizontal distribution of $p_0$ at the depth $H_c=60$ meters on 65th day of cold season (${k_v}>{k_v}^c, <w>>{w_c}$) of system 2, $p_0$ has higher sinking tendency, (**f**) Relation between $\langle w\rangle$ and ${k_v}^c$ using estimated data set, (**g**) Vertical distribution of $p_0$ of buoyant community ($k_p>w_p$) on 35th day of cold season of system 2 ($k_v<{k_v}^c$, $\langle w\rangle <w_c$), (**h**) Vertical distribution of $n_0$ of system 2 on 32nd day of cold season ($k_v<{k_v}^c, \langle w\rangle <w_c$), $p_0$ is buoyant in nature ($k_p>w_p$). The parameters values are mentioned in SI.

Figure 1c represents the vertical distribution of phytoplankton biomass of heavier community on the 115th day of the warmer season ($I_0=220\,W/m^2$) through a water column having depth $L_z=60$ meters in an aquatic medium of system 1. In contrast, vertical eddy diffusivity ($k_v$) is low, which still dominates horizontal eddy diffusivity ($k_v>k_h$) during low wind mixing period in the presence of higher grazing pressure. Under such circumstances, a surface bloom of the existing phytoplankton community has been observed on the 115th day while critical vertical eddy diffusivity dominates existing vertical eddy diffusivity ($k_v<{k_v}^c$) and zooplankton community is abundant on the surface zone (Fig. 2a). The figures (Figs. 1c, 2b) indicate that higher phytoplankton biomass provides ample food to grazers (zooplankton community), hence this aquatic food chain relation generates the possibility of bloom development of both of the species on the surface layer.

#### SB3: When phytoplankton community is buoyant in nature ($k_p>w_p$) and spatial average of water velocity is non-zero ($\langle u\rangle \ne 0,\langle v\rangle \ne 0,\langle w\rangle \ne 0$)

Figure 1d stands for the occurrence of surface bloom of buoyant species in an aquatic medium of system 2, in the presence of weak vertical eddy diffusivity ($k_h>k_v$) in an intense wind mixing period during the warmer season ($I_0=320\,W/m^{2}$) while $k_h>{k_h}^c$, $\langle u\rangle>{u_c},\langle v\rangle >{v_c}$ and grazing pressure does not hinder phytoplankton growth on surface water (Fig. 2a). The figure shows the vertical distribution of phytoplankton biomass ($p_0$) on the 39th day of the warmer season (late spring/ summer/ early autumn) through a water column having a depth of 60 meters. Figure 1d represents the vertical distribution of phytoplankton biomass ($p_0$) of the same community in a turbulent medium through a water column having a depth of 60 meters on the 39th day of summer season ($I_0=320\,W/m^{2}$) when $k_h<{k_h}^c$, $\langle u\rangle<{u_c},\langle v\rangle <{v_c}$ caused by the eventual stopping of the wind event. Therefore, in the presence of low grazing influence, intense wind mixing period on the horizontal layer gives rise to the fact $k_h>{k_h}^c$, $\langle u\rangle>{u_c},\langle v\rangle >{v_c}$, which initiates the process of surface bloom formation of phytoplankton community (Fig. 1e). In contrast, the eventual stopping of wind event under the same grazing pressure causes $k_h<{k_h}^c$, $\langle u\rangle<{u_c},\langle v\rangle <{v_c}$ and hinders surface bloom (Fig. 1d) and depending on the nature critical vertical eddy diffusivity ${k_v}^c$ and the spatial average of water velocity $\langle w\rangle$, nature of bloom formation or phytoplankton biomass accumulation at different layers varies accordingly (Fig.1d).

#### SB4: When phytoplankton community is heavier ($k_p<w_p$) in nature and spatial average of water velocity is non-zero

Figure 1f represents vertical distribution of phytoplankton biomass ($p_0$) of heavier species with higher sinking tendency in the presence of low grazing influence and weak vertical eddy diffusivity ($k_h>k_v$) while $k_h<{k_h}^c$, $\langle u\rangle<{u_c},\,\langle v\rangle <{v_c}$, $k_v<{k_v}^c$, $\langle w\rangle <w_c$ on 81th day of warmer season. Eventual stopping of the wind event during a thermally stratified period gives rise to the fact $k_h<{k_h}^c$, $\langle u\rangle<{u_c},\,\langle v\rangle <{v_c}$ along with $k_v<{k_v}^c$, which activates transportation of sinking biomass to upper layers in the absence of wind event and generates the possibility of bloom formation on the upper ocean in the presence of low grazing pressure.

### Bloom in higher depth (BHD)

#### BHD1: Phytoplankton community has higher sinking tendency ($w_p>k_p$)

Figure 1g represents the vertical distribution of phytoplankton biomass on the 50th day of the cold season ($I_0=160\,W/m^2$) through a water column having depth $L_z=60$ meters in an aquatic medium of system 2, where the phytoplankton community has higher sinking tendency ($w_p > k_p$) in the presence of high vertical eddy diffusivity satisfying $k_v>k_h$, since low surface irradiance $I_0=160$ causes less thermal stratification of surface water which triggers kinetic energy of water particles, hence vertical turbulent diffusion $k_v$ increases. It dominates the horizontal eddy diffusivity $k_h$ in the absence of a wind event. The intra-specific competition among herbivorous zooplankton classes for the same food sources remains high, and the grazing and assimilated grazing coefficient remains low. This is the case where a lack of thermal stratification triggers vertical eddy diffusivity to initiate the possibility of phytoplankton bloom formation at higher depths in the presence of low grazing pressure.

#### BHD2: When phytoplankton community is buoyant in nature ($w_p<k_p$)

Figure 1h represents the vertical distribution of phytoplankton biomass of buoyant community on the 70th day of the cold season ($I_0=160$) through a water column having depth $L_z=60$ meter in the presence of high vertical eddy diffusivity ($k_v$) satisfying ${k_v}>k_h$ (which happens when no wind event affects surface waves of turbulent flow) in a turbulent medium ($\langle u\rangle \ne 0,\langle v\rangle \ne 0,\langle w\rangle \ne 0,$ where $\langle w\rangle >\langle u\rangle ,\langle v\rangle$). According to this figure, when critical vertical eddy diffusivity is crossed (${k_v}^c<k_v$) and vertical water advection dominate its critical value ($\langle w\rangle >w_c$) in the presence of low grazing influence ($g_z=0.4$), species sink to higher depth and gets accumulated at the bottom layer ($L_z=60<D_c$, SI Table 2) and eventually bloom occurs over there.

*Note:* Relation between controlling factors and depths are provided in SI Tables 1, 2 of system 1 and system 2 respectively.

## Discussion

Phytoplankton experience turbulence as an instantaneous, linearly varying fluid velocity across the cell body^32,33^, and biomass distribution^34^. Nonmotile phytoplankton such as some diatom and cyanobacteria species orient in hydrodynamically favorable directions to displace over large distances^35^ by either positively or negatively varying their buoyancy through different mechanisms such as gas vesicles, lipid storage, the exchange of heavy ions with their surroundings or by forming chains that modify their sinking rates^36–38^. Even though it is energetically demanding, motility is advantageous in a world where resources (nutrients) are heterogeneously distributed^39^. The majority of the phytoplankton classes are flagellated and, therefore, motile. Marine pelagic plankton such as ciliates, flagellates, and copepods exhibit a wide variety of motility patterns such as straight line and helical swimming, darting motion, etc. Cells move to new locations to seek more favorable environments or to interact with other organisms. Along with other variety of cues, including their encounter with other individuals (prey, a predator, or a mate), or environmental conditions such as light^40^, temperature, etc., these complex motility patterns are behavioral responses of plankton, which is controlled by turbulence^41^, which vary with regional and seasonal changes. Water turbulence significantly affects the distribution of phytoplankton^42^, and this leads to the concept that the formation of phytoplankton patches depends on the balance between phytoplankton growth rate and nature of eddy diffusivity, which controls the diffusion of the water^43^.

For any aquatic medium, the occurrence of bloom depends on how eddy diffusivity drives the distribution and dynamics of existing communities and how the existing community successfully dominates overcoming all hindrances. In order to investigate the reasons behind such occurrences with seasonal variation, we have focused on five components in our numerical investigation, namely,

(i) nature of dominating phytoplankton community,
(ii) horizontal and vertical eddy diffusivity,
(iii) spatial average of water velocity,
(iv) critical depth ($D_c$), and
(v) grazing impact.

We now consider the case of occurrence of bloom at a higher depth $H_c$ for predefined systems considering two scenarios,

(i) when vertical eddy diffusivity ($k_v$) is the only dominating component in system 1;
(ii) when the vertical component of the spatial average of water velocity dominates over the corresponding horizontal component ($\langle w\rangle >\langle u\rangle ,\langle v\rangle$) and vertical eddy diffusivity dominates horizontal eddy diffusivity (${k_v}>{k_h}$) in system 2.

We now address seven major research questions, which are the focus of our work.

*The first question which we like to address is why phytoplankton bloom in higher depth is so common in nature during the winter season, and when can bloom also be developed in the upper ocean or surface water in the same season?*

During the cold season, due to less thermal stratification of surface water for both predefined systems, the stability of the water column gets disturbed, causing an increment in vertical turbulent diffusion in the less stratified layer. Besides, externally induced artificial mixing causes an imbalance in the vertical velocity distribution of stationary flow and generates a turbulent environment, which triggers vertical eddy diffusivity $k_v$. As $k_v$ gets affected, vertical mixing gets affected as well; as a result, the process of stabilization gets started to restore the system’s stability through the transportation of biomass carried within the water column. While this stabilization process continues, phytoplankton biomass gets shifted from a highly concentrated zone to a less dense zone. Under such circumstances, considering the grazing impact to be low, several scenarios may arise regarding the occurrence of bloom depending on the nature of overall horizontal pull (triggered by $k_p,k_h,\langle u\rangle ,\langle v\rangle$) and vertical pull (triggered by $w_p,k_v,\langle w\rangle$) on phytoplankton biomass ($p_0$) available on surface water, which are determined from the nature of dominating phytoplankton communities and variation in the nature of aquatic environment with seasonal changes.

For turbulent medium (system 2), in the presence of high vertical mixing caused by higher vertical eddy diffusivity $k_v$, when the vertical component of the spatial average of water velocity ($\langle w\rangle$) dominates the corresponding horizontal component ($\langle u\rangle ,\langle v\rangle$), being influenced by the change in the direction of the oceanic current, phytoplankton bloom of any community irrespective of whether it is buoyant ($k_p>w_p$) or its buoyancy gets dominated by its higher sinking tendency ($k_p<w_p$), can occur at a higher depth $H_c$ where sinking species gets stuck. Phytoplankton growth remains higher than phytoplankton loss at $H_c$, that is, $H_c<D_c$, depending on the nature of vertical pull and grazing influence. If the dominating community tends to sink ($w_p>k_p$, case BHD1), when $\langle w\rangle$ and vertical eddy diffusivity ($k_v$) dominate over horizontal components ($\langle u\rangle ,\langle v\rangle ,k_h$) in system 2, overall vertical pull on surface biomass increases. In such a scenario, dominating factors $w_p,\,k_v,\,\langle w\rangle$ take charge of the system’s stability and the bloom’s interconnected nature, provided that the grazing impact does not influence phytoplankton growth at the depth $H_c$ where sinking species accumulate. If the overall growth of phytoplankton communities at $H_c$ does not get annihilated by total loss over there, which happens when $H_c<D_c$, then the occurrence of bloom at $H_c$ depends only on the nature of dominating vertical velocity and diffusion parameters (Fig.1(g)).

On the other hand, in the absence of a spatial average of water velocity, the chance of bloom formation at a higher depth ($H_c$) for system 1 depends on the transportation rate of phytoplankton biomass along with the nutrient and the possibility of survival of sinking phytoplankton species at that depth ($H_c$). In such a case, vertical eddy diffusivity ($k_v$) plays a crucial role in bloom development at $H_c$.

Stronger vertical pull influences the chances of bloom initiation at higher depths, but only higher values of vertical eddy diffusivity ($k_v$), $\langle w\rangle$, and $w_p$ are not sufficient enough for causing bloom at higher depth if mixing of biomass is limited to upper surface within the water column. This is possible in the presence of a high value of $\langle w\rangle$, which induces a higher sinking tendency of dominating community, causing phytoplankton biomass to get distributed throughout the water column. However, the lack of vertical eddy diffusivity ($k_v$) cannot provide the essential requirement of nutrients to the existing species at higher depths, which hinders species growth, implying that bloom cannot occur at higher depths. Higher vertical eddy diffusivity ($k_v$) along with $\langle w\rangle$ being less than corresponding critical threshold values ${k_v}^c$, $w_c$ causes most of the phytoplankton biomass to get heterogeneously and non uniformly distributed at different layers within the upper zone of the water column, which causes initiation of bloom at some of those layers, where grazing impact remains negligible and does not hinder phytoplankton growth. Figure 2c represents such a situation where low surface irradiance causes high vertical eddy diffusivity during the cold season in the presence of a high spatial average of water velocity along the vertical direction, caused by an exchange of oceanic current when higher sinking tendency of existing phytoplankton community dominates over its buoyancy ($w_p>k_p$). Current values of dominating factors ($k_v,\langle w\rangle$) being less than their critical values cause bloom ($H_c$, Fig. 2c ($0\le H_c\le 5, 10\le H_c \le 20$)) or higher phytoplankton biomass (Fig. 2d ($0\le H_c\le 5$)) at upper layers within the euphotic zone. One of these factors might be the reason for phytoplankton bloom development in the central Cantabrian Sea during late winter, where a slight increment in water temperature results in the reduction of vertical eddy diffusivity $k_v$^14^. Besides, several phytoplankton patches of density varying from low (dark green) to high (yellow) observed in horizontal distribution (Fig. 2e) stand for the intermittency in the phytoplankton distribution of heavier communities during the cold season. This successfully explains observed intermittency in distributional dynamics of heavier species *“Skeletonema Costatum”* at a higher depth of 50 meters of Tokyo Bay in Dec 2006 and Feb 2008^8^.

Numerically, it has been observed that critical value ${k_v}^c$ varies inversely with $\langle w\rangle$ irrespective of whether the community is buoyant ($k_p>w_p$) or has a higher sinking tendency ($w_p>k_p$) (Fig.2(f)). Therefore, under the influence of the same circumstantial factors, the type of aquatic medium determined by the nature of water velocity decides the nature of phytoplankton bloom. Hence, in the presence of a high spatial average of water velocity along vertical direction satisfying $\langle w\rangle >w_c$ for the turbulent environment (system 2), ${k_v}\,(>{k_v}^c)$ can cause bloom at higher depth ($H_c$) of water column (Fig. 1g, $H_c=L_z=60$ meters), same $k_v$ in the absence of $\langle w\rangle$, being extremely less than ${k_v}^c$ in system 1, cannot transport excessive phytoplankton biomass from surface to deeper layers (Fig. 2d) and most of the phytoplankton biomass gets distributed within the upper surface of euphotic zones attached to the surface layer of water column (Fig. 2d), obstructing phytoplankton bloom initiation at higher depths. Therefore, if grazing by herbivorous zooplankton does not exert any negative impact on phytoplankton biomass available on surface water, then lack of transportation of excessive biomass from surface to bottom layer causes accumulation of phytoplankton biomass on the upper layer, which eventually generates the possibility of the occurrence of bloom at that upper layer during the winter-spring season for aquatic mediums like the lake, pond, and calm marine ecosystem, where wind velocity or exchange of seasonal currents are not the disturbing factors to ruin the calmness. On the other hand, if critical turbulence itself gets dominated by existing vertical eddy diffusivity ($k_v>{k_v}^c$) and along with that, dominating component of the spatial average of water velocity remains higher than its critical value ($\langle w\rangle >{w_c}$) for turbulent medium (system 2), phytoplankton bloom can occur at a higher depth (say, $H_c$) or at the bottom layer of the water column ($L_z$) (Fig. 1g), where intrinsic growth of phytoplankton is positive due to low grazing.

Though the bloom development at higher depth is triggered by higher vertical pull initially, it is not sufficient to cause bloom at deeper zones if the sinking rate of the existing phytoplankton community is negligible and the distribution of phytoplankton biomass is limited to the upper layers of the euphotic zone. This happens when existing species is almost buoyant ($w_p\approx 0$), and it is the only dominating community during that season. Under such circumstances, higher vertical eddy diffusivity in the presence of a high spatial average of vertical water velocity causes transportation of some buoyant species into deeper levels for turbulent medium (system 2). However, in the absence of sufficient vertical pull, most phytoplankton biomass remains non-uniformly distributed at upper layers attached to surface water. Therefore, for system 2, even if the grazing impact remains low on surface water, such a non-intermittent assortment of existing biomass of dominating phytoplankton community within the upper layers of euphotic zone and sinking loss of existing community, both caused by vertical pull, hinder surface bloom. For such cases, depending on circumstantial factors, there exist critical threshold values of vertical eddy diffusivity (${k_v}^c$) and the spatial average of water velocity ( $w_c$). When existing vertical eddy diffusivity $k_v>{k_v}^c$ along with $\langle w\rangle >w_c$ for system 2, transportation of excessive mass to deeper level triggers chances of bloom at a higher depth $H_c$ or at the bottom layer $L_z$ (Fig. 1h), provided both $H_c$ or $L_z$ are less than critical depth $D_c$. However, if $k_v<{k_v}^c$ and $\langle w \rangle <w_c$, then enough phytoplankton biomass of the buoyant community cannot be shifted to a deeper level to initiate bloom; instead, most of the phytoplankton biomass gets heterogeneously distributed within upper mixed layers and some sink to a specific small layer within the euphotic zone (Fig. 2g). Besides, this condition also triggers an upwelling event to transmit deep nutrient-rich water to the upper layer (Fig. 2h), which eventually causes nutrient abundance on the surface and upper oceanic layers generating the possibility of a phytoplankton bloom on surface water or in upper oceanic layers within the euphotic zone, where sinking phytoplankton biomass gets accumulated ($0\le H_c\le 20$m as observed in Fig. 2g), and does not get negatively influenced by grazing pressure. This explains the fact of phytoplankton spring bloom initiation near-surface water of the temperate North Atlantic Ocean due to relaxation in turbulence profile^11^. It also supports the fact that phytoplankton bloom developed at a depth of nearly 10 meters of the mouth of the Arakawa river in May 2011^8^.

**Figure 3:**
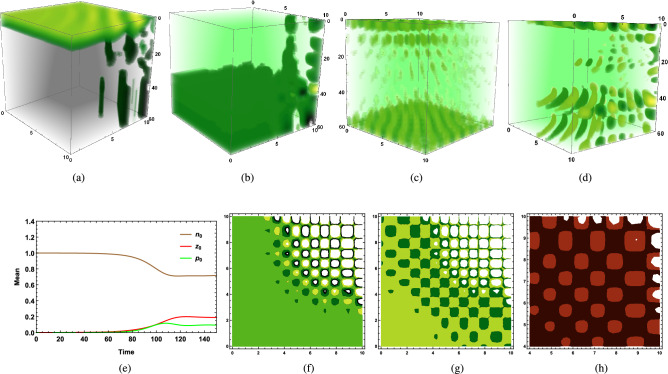
(**a**) Vertical distribution of $p_0$ of buoyant community on t=35th day of cold season of system 1 ($k_v<{k_v}^c$), (**b**) Vertical distribution of $p_0$ of buoyant community on t=40th day of cold season of system 1 ($k_v>{k_v}^c$, (**c**) Vertical distribution of $p_0$ on 95th day of cold season of system 1 ($k_v>{k_v}^c$), $p_0$ has higher sinking tendency ($w_p>k_p$), (**d**) Vertical distribution of $p_0$ on 80th day of summer season of system 1 when $p_0$ is buoyant ($k_p>w_p$, $k_v>{k_v}^c$), (**e**) Time variation of $p_0$ and $z_0$ on surface water ($k_v<<{k_v}^c$) of system 1 (summer season), $p_0$ is heavier ($w_p>k_p$), (**f**) Horizontal distribution of $n_0$ on surface layer on 62nd day of summer season (${k_h}>{k_h}^c, \langle u\rangle>{u_c}, \langle v\rangle >{v_c}$) for system 2, $p_0$ is buoyant in nature ($k_p>w_p$), (**g**) Horizontal distribution of $n_0$ on surface layer on 71th day of summer season (${k_h}>{k_h}^c, \langle u\rangle<{u_c}, \langle v\rangle <{v_c}$) of system 2, (**h**) Horizontal distribution of $p_0$ on 70th day of summer season, $p_0$ is buoyant in nature ($k_p>w_p$, ${k_h}>{k_h}^c, \langle u\rangle>{u_c}, \langle v\rangle >{v_c}$) (red tide formation). The parameters values are mentioned in SI.

Critical vertical eddy diffusivity ${k_v}^c$ increases as $\langle w\rangle$ decreases (Fig. 2f), as a result of which system 1 requires higher $k_v$ than that of the system 2 having non-zero $\langle w\rangle$ (see SI Tables 1, 2). Therefore, under the influence of the same circumstantial factors, existing vertical eddy diffusivity crossing its critical threshold value ${k_v}^c$ transports sufficient biomass from the surface towards bottom layers in the presence of a high spatial average of vertical water velocity $\langle w\rangle$ greater than its critical value in system 2. However, the same vertical eddy diffusivity, in the absence of spatial average of vertical water velocity ($\langle w\rangle =0$) for system 1, remains less than critical value ${k_v}^c$, which creates hindrance in bloom formation at higher depth due to the obstruction of such transportation of biomass (Fig. 3a). Besides, when it comes to the occurrence of bloom in a deeper zone of system 1, vertical eddy diffusivity (being higher than its critical value) triggers the chance of bloom initiation at a deeper level $H_c$ (Fig. 3b), provided ${H_c}$ is less than $D_c$ with low grazing. All the stated facts well explain the reason for phytoplankton bloom in higher depths of seas, oceans, or lakes during the cold season (Figs. 1g,h, 3b,c), which has been observed in deep sea waters of North Arabian Sea during the winter season (Jan–March)^13^.

Numerical results under cases BHD1 and BHD2 also explain why surface bloom in winter is not so common in nature for system 2. First, lack of surface irradiance blocks phytoplankton growth on the surface mixed layer. Besides, whatever the net growth is, extreme surface water stratification causes high vertical eddy diffusivity with $k_v>{k_v}^c$ when $\langle w\rangle >w_c$, influenced by the exchange of seasonal currents. This results in shifting most of the phytoplankton biomass to a deeper level (Fig.1g,h), which ultimately blocks winter bloom on the surface mixed layer for most cases. As the winter season passes and early spring occurs, surface irradiance increases, which causes a slight increment in net productivity, resulting in a thicker phytoplankton layer on surface water that absorbs most of the incident solar irradiation. This explanation is valid for any aquatic environment regarding winter bloom formation at higher depths^8^.

*Our next question is what triggers surface bloom formation for the almost calm Baltic Sea in spring season*?

The study suggests that stratification of the water column is generally a prerequisite for most dinoflagellate blooms to develop in temperate areas^44,45^. In Baltic Sea, dinoflagellates are more sensitive to hydrographic conditions and climate fluctuation than to nutrient input in the Baltic sea on bloom onset^44^. Besides, experimental study has suggested that since the 1980s the nutrient loads to the Baltic Sea have been continuously decreasing^44^. According to this study^46^, an increase in thermal stratification can influence species-specific dinoflagellate distribution, behavior, and survival. Increased thermocline strength may decrease mixing between deep nutrient-rich and surface nutrient-depleted waters, leading to a decrease in surface water productivity^47^. However, stratification seems to favor species that can access the nutrient-rich water layers by the process of diel vertical migration (DVM), and the formation of algal blooms in low-nutrient surface waters is therefore possible^48^. Our study captures the dinoflagellates blooming on surface water in the spring season (Feb/March) in the southern Baltic Sea caused by water warming in the absence of extreme wind events^10^. The Baltic Sea is calm primarily during spring and summer, falling under system 1. As spring or summer arrives, water temperature increases due to an increment in irradiation, which reduces vertical eddy diffusivity $k_v$. Such reduction causes $k_v$ to be less than its critical threshold value ${k_v}^{c}$. Hence, the transportation rate of mass from the surface to the bottom layer decreases for this almost calm marine environment in the absence of any wind event or exchange of oceanic current. Most phytoplankton species remain distributed on the upper layer within the water column. Therefore, species that have already sunk to higher depths and previously caused winter bloom at the depth $H_c\,(< D_c)$ die because of a lack of photosynthesis caused by a lack in the transportation of essential elements required for this process. Hence, the chance of blooming in higher depths reduces. In case the species is buoyant in nature, for example dinoflagellates, less sinking causes most of its biomass to accumulate on surface layer, which triggers the initiation of dinoflagellates surface bloom during early spring (Fig. 1a). In fact, excluding Baltic Sea, this explanation is valid for any aquatic medium under system 1 regarding the occurrence of surface bloom during early spring. Unless artificially induced vertical mixing generates instability in the water column and initiates the transportation of higher phytoplankton biomass and other ingredients from the surface to the bottom of the water column during the summer season, phytoplankton bloom usually occurs on the surface layer due to the impact of high thermal stratification of surface water.

*Our third query, why is the surface bloom of the phytoplankton community so common in nature during summer, spring, or autumn?*

The critical turbulence hypothesis proposes that a surface bloom can start under two different circumstances, (i) if the vertical eddy diffusivity is weak enough in the well-lit surface water for phytoplankton to receive sufficient light before being properly mixed beneath the critical depth or (ii) an intensely mixed surface layer shoals exposes phytoplankton to favorable light conditions^49,50^, which is possible when strong wind mixing period drives surface current to produce high horizontal eddy diffusivity ($k_h$) in the presence of a high spatial average of the horizontal component of water velocity ($\langle w\rangle$), which leads the existing community to get exposed to a favorable light and nutrient-rich zone.

As far as system 1 (lake, pond, or calm marine environment) is concerned, the absence of surface winds cannot trigger internal waves to inherit any horizontal turbulence. Hence, vertical eddy diffusivity is the only controlling factor for such a system, which acts on turbulent mixing and determines the nature of the planktonic distribution. When externally induced artificial mixing does not cause an imbalance in the nature of stationary flow, variation of vertical eddy diffusivity depends mostly on water density and temperature. During late spring, summer, and early autumn, high incident solar irradiance causes extreme thermal stratification to subjugate the density of water among water layers, resulting in reduced vertical eddy diffusivity $k_v$. Under such circumstances, the nature of phytoplankton distribution varies depending only on the nature of critical vertical eddy diffusivity ${k_v}^c$. For system 1, zero contribution of the spatial average of water velocity to turbulent mixing requires ${k_v}^c$ to be sufficiently higher for bloom initiation at higher depths, mostly when the buoyancy of existing community ($k_p>w_p$) drives most of the primary producers to float away and spread over a larger domain horizontally (Fig.1(b)), than diving to deeper levels. Since $k_p>w_p$ influences the horizontal pull, it stirs up the initiation of surface bloom in the presence of low grazing impact (Fig.1(a)). When existing vertical eddy diffusivity ${k_v}\,(<{k_v}^c$) prevents the transportation of essential ingredients like temperature-induced warm water, nutrient, and several other abiotic factors required for photosynthesis, it accelerates the chance of initiation of surface bloom by causing no reduction in phytoplankton growth. This result explains why buoyant species float upwards and form dense surface blooms during weak mixing^51–54^. This result also mimics the fact that the buoyant phytoplankton community “*Microcystis*” developed a surface bloom during July 1992 in Lake Nieuwe Meer^15^.

Nevertheless, for stationary flow, phytoplankton bloom of buoyant class can also be initiated in an arbitrarily deeper layer (Fig. 3d) above critical depth in spring, early summer, or autumn season if externally induced artificial mixing triggers vertical eddy diffusivity to cross its critical threshold value. Then, existing surface biomass gets transported to a higher depth $H_c$ or to the bottom layer $L_z$ until its sinking to a higher depth of the water column is hindered by the water body’s resilience. Then bloom can occur at that higher depth when ${k_v}>{k_v}^c$, provided ${H_c}\,\text {or}\,L_z<D_c$ (Fig. 3d). This explains the fact of the occurrence of bloom of “*Microcystis*” at higher depth in Lake Nieuwe Meer during initial days in the presence of artificial mixing^15^. In fact, the hypothesis stated in^49^ is supported by our numerical findings and the corresponding real-life occurrence regarding phytoplankton spring bloom initiation in an arbitrarily deep layer.

*We now explain how* ${k_v}<{k_v}^c$ *controls the nature of the bloom of existing phytoplankton species if its sinking tendency dominates its buoyancy in system 1?*

Fundamentally speaking, the heavier phytoplankton community sinks deeper, accelerating in the presence of higher vertical eddy diffusivity during the cold season. In general, strong vertical eddy diffusivity facilitates mixing, intensifying the possibility of bloom development of the heavier community at higher depth during that period (Fig. 1g). Though the higher sinking tendency of the heavier community like diatom triggers species sinking to deeper levels, the nature of vertical mixing sometimes controls and stops such sinking, depending on the nature of horizontal mixing and thermal stratification of water during the low wind mixing period in spring, early summer or autumn season. While wind speed does not take part in phytoplankton distribution during the thermally stratified period at the beginning of the spring season in lake water (system 1), increment in water temperature hinders increment in the dominating factor $k_v$ while the spatial average of the vertical velocity of water is zero. This results in $k_v<{k_v}^c$, which obstructs the transportation of surface biomass, including nutrients, several organic factors, and phytoplankton biomass from the surface to the bottom layer. Besides, the occurrence of vertical upwelling event influenced by the fact $k_v<{k_v}^c$, causes vertical transmission of nutrients and sinking phytoplankton biomass to the surface layer, as a result of which deep nutrient-rich water reaches the surface level, which provides an ample supply of nutrient to the existing phytoplankton community that enhances phytoplankton productivity and growth. An abundance of nutrients (Fig. 3e) and high water temperature accelerate phytoplankton productivity and growth. Low wind mixing generates low horizontal eddy diffusivity $k_h$, which causes biomass to get distributed over surrounding regions and reduces the pressure of phytoplankton biomass on the surface layer. Hence, high productivity and spreading of phytoplankton biomass initiates surface bloom development (Fig. 1c). Under such circumstances, phytoplankton bloom gets initiated for a specific time, even when the impact of high grazing pressure causes zooplankton abundance (Fig. 2b). In fact, this result mimics the event of the occurrence of bloom of both phytoplankton class (diatom with higher sinking tendency in shallower lakes) and zooplankton class (*Cladocerans* dominated by the *Daphnia hyalina-galeata* complex) as mentioned in^16^.

*Our next analysis answers the question what drives surface bloom formation of buoyant phytoplankton community in a turbulent marine environment?*

Wind-induced horizontal turbulence is one of those critical factors for developing surface bloom in system 2. In a turbulent environment, wind-driven horizontal currents deliver deep nutrient-rich water to the surface photic layers. Also, in the presence of weak vertical eddy diffusivity during the warmer season, wind-induced strong horizontal eddy diffusivity ($k_h$) triggers horizontal mixing, which along with a higher spatial average of horizontal water velocity ($\langle u\rangle ,\langle v\rangle$) of system 2 lead all plankton bodies, nutrients, warmer water to a comparatively stationary surface zone. A fair wind is required to cause such transportation since only higher values of horizontal water velocity and turbulence are not sufficient enough to initiate surface bloom. Corresponding to that, we have considered there exist critical values ${k_h}^c,{u_c},{v_c}$ (say) of horizontal eddy diffusivity and the spatial average of horizontal water velocity component, respectively, which decide whether the existing surface wind is appropriate to trigger the surface bloom. If existing horizontal eddy diffusivity dominates its critical value, while the spatial average of horizontal water velocities have already crossed their critical values, horizontal transportation of phytoplankton biomass along with required ingredients from dense, turbulent zone to stationary zone (${k_h}>{k_h}^c$, $\langle u\rangle>{u_c}, \langle v\rangle >{v_c}$) generates the possibility of surface bloom initiation (Fig. 1e) in the presence of low grazing pressure on surface water.

On the contrary, whenever this condition is violated by the nature of wind (${k_h}<{k_h}^c$, $\langle u\rangle<{u_c}, \langle v\rangle <{v_c}$), lack of horizontal transportation of surface biomass makes it heavier to dominate water resilience and sink into slightly deeper zone getting influenced by existing vertical turbulence. However, weak vertical mixing caused by weak vertical eddy diffusivity ($k_v<{k_v}^c$) during such a thermally stratified period with $\langle w\rangle <w_c$ cannot drive phytoplankton species deeper; instead, they sink to a particular layer ($H_c$) within the upper zone of the euphotic layer where nutrients and light are sufficiently available for the continuation of photosynthesis to maintain marine food web. This results in higher phytoplankton biomass at that layer (Fig. 1d, $H_c=20$ m) with negligible grazing.

Generally, the effect of turbulent current is different for different phytoplankton species. In particular, the conditions ${k_h}>{k_h}^c$, $\langle u\rangle>{u_c}, \langle v\rangle >{v_c}$ stand for the bloom of those species which are buoyant and capable of growing in a highly turbulent environment. Wind-induced horizontal turbulence drives nutrients along with all patches of buoyant species to an exceptionally stable surface layer, where the currents are more or less steady, and vertical turbulence is critically weak due to extreme thermal stratification triggered by intense solar irradiation during summer, late spring or early autumn season and nutrient density keeps on growing on the surface layer with time (Fig . 3f$\,\rightarrow$ g). Therefore, when grazing pressure does not exert any negative impact on phytoplankton on surface water (Fig. 2a), the surface bloom of the existing buoyant community gets developed in nutrient-rich saline water (Fig. 1e). This might possibly be the reason behind several nonmotile flagellate classes, like *“Alexandrium Tamarense”*, forming red tides (Fig. 3h) (flagellates bloom) in Mikawa Bay of Japan in a severe diffusive condition with horizontal turbulence $k_h$ is of order $10^2\,m^2\,s^{-1}$ and vertical turbulence $k_v$ is of order $10^{-5}\,m^2\,s^{-1}$ in spring, 1991 (28th March–22nd April)^9^. Whenever the growth of turbulent driven species is immensely responsive to such an environment (${k_h}>{k_h}^c$, $\langle u\rangle>{u_c}, \langle v\rangle >{v_c}$), surface bloom or red tides (Fig. 3h) of motile buoyant classes gets formed as well in the presence of low grazing impact. Such a scenario explains the red tide formation of several motile, buoyant flagellates like *“Ceratium Furca”* (July 1990: Summer), *“Ciliate”*, *“Mesodinium Rubrum”* (November 1991: Autumn), etc. in Mikawa Bay, Japan during several periods from April 1989 to Dec 1991^9^.

**Figure 4:**
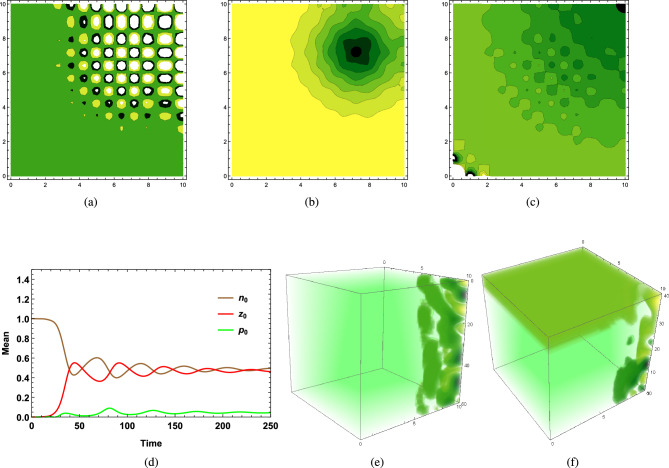
(**a**) Horizontal distribution of $n_0$ on surface layer on 64th day of summer season (${k_h}>{k_h}^c, \langle u\rangle>{u_c}, \langle v\rangle >{v_c}$) of system 2, $p_0$ is heavier in nature ($k_p<w_p$), (**b**) Horizontal distribution of $n_0$ on surface layer on 70th day of summer season with same conditions, (**c**) Horizontal distribution of phytoplankton variance $\langle p'^2\rangle$ on 75th day of warmer season of system 2 ($k_h>{k_h}^c, \langle u\rangle >u_c,$ $\langle v\rangle > v_c$), $p_0$ is heavier ($w_p>k_p$), (**d**) Time variation of $p_0$ and $z_0$ in summer season of system 2 ($k_v<{k_v}^c$), $p_0$ is heavier, (**e**) Vertical distribution of $p_0$ of heavier community on 168th day of summer season of system 2 ($k_h>{k_h}^c, \langle u\rangle>u_c, \langle v\rangle v_c, k_v>{k_v}^c$), (**f**) Vertical distribution of $z_0$ on 168th day of summer season ($I_0=220\,W/m^2$) of system 2, with same condition. All the figures are in the presence of high grazing influence. The parameters values are mentioned in SI.

*Now, we would like to address what happens to the occurrence of surface bloom while dominating class has a higher sinking tendency* ($w_p>k_p$) *due to heavier size in a wind-induced turbulent flow?*

**Figure 5:**
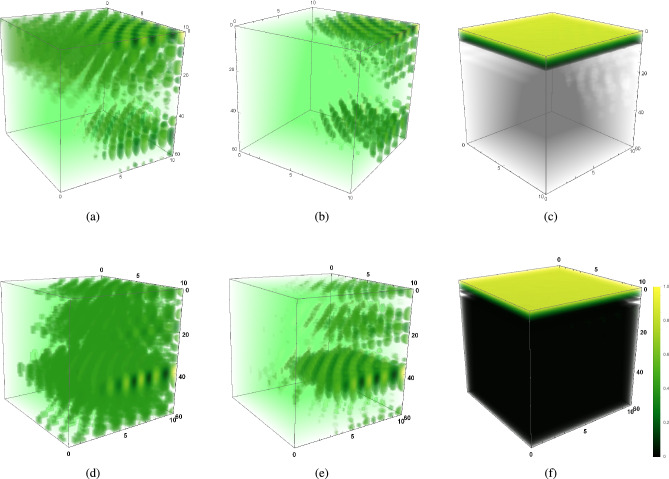
Bloom cycle of heavier community of $p_0$ as $k_h<{k_h}^c, \langle u\rangle<u_c, \langle v\rangle <v_c$ shifts to $k_h>{k_h}^c, \langle u\rangle>u_c, \langle v\rangle >v_c$ in the presence of low grazing. (**a**) Vertical distribution of $p_0$ on 50th day of cold season of system 2 ( $k_h<{k_h}^c, \langle u\rangle<u_c, \langle v\rangle <v_c$), (**b**) Vertical distribution of $p_0$ on 53rd day of cold season with same conditions, (**c**) Vertical distribution of $p_0$ on 62nd day of cold season of system 2 ($k_h>{k_h}^c, \langle u\rangle>u_c, \langle v\rangle >v_c$), (**d**) Vertical distribution of $p_0$ on 65th day of cold season of system 2 ( $k_h<{k_h}^c, \langle u\rangle<u_c, \langle v\rangle <v_c$), (**e**) Vertical distribution of $p_0$ on 70th day of cold season of system 2 with same conditions, (**f**) Vertical distribution of $p_0$ on 75th day of cold season of system 2 ($k_h>{k_h}^c, \langle u\rangle>u_c, \langle v\rangle >v_c$). The parameters values are mentioned in SI.

$w_p$ triggers vertical pull on available biomass of heavier community; hence, ensuring the possibility of surface bloom development requires intense wind-driven horizontal eddy diffusivity, which will transport heavier phytoplankton body to a dense nutrient-rich zone to dominate its sinking by the resilience of the dense water body of that zone. Generally, during a thermally stratified period, vertical eddy diffusivity reduces and reaches beneath its critical level ($k_v<{k_v}^c$), as a result of which existing $k_v$ cannot transport excessive mass from surface to bottom layer; instead, it initiates upwelling of water, which transports nutrient-rich water of bottom layers to surface level. As this happens, an increment in water density enhances the chance of water column instability, which triggers two related events under two different circumstances. First, during intense wind mixing period, if existing horizontal eddy diffusivity dominates its critical value ($k_h>{k_h}^c$) along with $\langle u\rangle>{u_c},\,\langle v\rangle >{v_c}$), then this drives extreme dense water along with existing phytoplankton biomass from highly dense, turbulent zone to approximately stable region, where dense water and overall biomass spread over a larger domain to maintain system stability. As the overall biomass of nutrients and phytoplankton gets assembled in a stable domain, the resilience of dense water bodies eventually obstructs species from sinking to higher depths. Besides, in the presence of low grazing impact, increment in nutrient biomass with time provides an ample supply of nutrients (Fig. 4a$\rightarrow$b), here the light yellowish shaded zone stands for higher nutrient biomass in Fig.4b compared to Fig. 4a, to the existing phytoplankton community on the surface zone. Additionally, inadequate light availability on the surface layer triggers species productivity which eventually strengthens the possibility of surface bloom (Fig. 5c,f). Such bloom of heavier species, namely, dinoflagellates and diatoms, has been observed in the southern Baltic Sea in Feb/March^10^. The above facts also explain the event of spring bloom initiation on the surface water of the North Atlantic Ocean during a thermally stratified period associated with strong westerly winds in winter-spring^55^.

It is highly unrealistic for a wind-driven turbulent medium (system 2) in the absence of any intense exchange of oceanic currents (implying $\langle w\rangle \approx 0$) to have bloom at higher depth, since during wind mixed and stratified period $k_h$ remains of order $10^2$, therefore, the condition $k_v>{k_v}^c$ to hold, $k_v$ needs to be of order $10^{15}$ to cross ${k_v}^c$ as it has numerically been observed for $k_h=9\times 10^3$, ${k_v}^c \ge 2.3658\times 10^{14}$, which is practically impossible as thermal stratification does not let $k_v$ to increase this much unless the system is almost stationary where $k_h$ will be critically low. Therefore, for most cases, even if $k_h<{k_h}^c$ causes an increment in $k_v$, it remains less than ${k_v}^c$ unless the turbulency is gone due to eventual stopping in wind event during a thermally stratified period in the absence of any intense exchange of oceanic currents. Whenever this happens, though heavier weight causes species to dive deeper, the fact $k_v<{k_v}^c$ along with $\langle w\rangle <w_c$ hinders transportation of surface biomass to a deeper level, and whenever some species sink due to sinking nature, they get shifted to the upper layer and remains heterogeneously distributed over there (Fig. 1f). In particular, variation in the nature of the horizontal eddy diffusivity and water velocity generates the possibility of two different circumstances, (i) $k_h>{k_h}^c,\,\langle u\rangle>{u_c},\,\langle v\rangle >{v_c}$, (ii) $k_h<{k_h}^c,\,\langle u\rangle<{u_c},\,\langle v\rangle <{v_c}$, which can be biologically interpreted as the impact of the episodic wind event. When wind hits the ocean surface, sudden acceleration in the horizontal eddy diffusivity and water velocity results in $k_h>{k_h}^c,\,\langle u\rangle>{u_c},\,\langle v\rangle >{v_c}$ and $k_v<{k_v}^c$, which initiates a bloom cycle. However, in between two wind events, a particular gap exists that causes a reduction in horizontal eddy diffusivity and velocity distribution of oceanic flows. Such reduction causes $k_h<{k_h}^c,\,\langle u\rangle<{u_c},\,\langle v\rangle <{v_c}$, which makes the system unstable during the bloomy period, and species starts to sink again. Under such circumstances, depending on the nature of $k_v,\,\langle w\rangle$ and driven by the fact whether ($k_v<\text {or}>{k_v}^c$, $\langle w\rangle <\,\text {or}\,>w_c$), species remains homogeneously distributed in the upper ocean due to upwelling of water ($k_v<{k_v}^c$, $\langle w\rangle <w_c$ (Fig. 1d,f)) or they dive deeper due to $k_v>{k_v}^c$, $\langle w\rangle >w_c$ (Fig.5a,b). If another episodic wind event hits the ocean surface during this process, then it again activates horizontal components to cause $k_h>{k_h}^c,\,\langle u\rangle>{\langle u\rangle }^c,\,\langle v\rangle >{\langle v\rangle }^c$ and $k_v<{k_v}^c$, $\langle w\rangle <w_c$. This breaks the cycle of species sinking, and the bloom cycle begins again similarly (Fig.5d–f).

This bloom cycle has been numerically captured in Fig. 5, where phytoplankton bloom has been observed on surface water and the upper layer of the euphotic zone on the 62nd and 75th day of the summer season in two weeks. For the first bloom, starting from the 50th day onwards, the sinking of heavier species to deeper levels eventually starts to decrease, and for the second bloom, starting from the 65th day onwards, the sinking of heavier community decreases, which happens due to variation in the nutrient density on surface water in the presence of episodic wind event. This is observed numerically in the case SB4 where nutrient density on the surface layer is higher on the 70th day (Fig.4b) than that on the 64th day (Fig.4a), which makes water nutrient-rich and provides ample nutrients required for photosynthesis to the existing phytoplankton community on surface layer resulting enhancement in phytoplankton community growth. Generally, the sinking rate of heavier classes like diatom increases with nutrient stress^56^. As a result, an inadequate supply of nutrients driven by the fact $k_h>{k_h}^c,\,\langle u\rangle>{u_c},\,\langle v\rangle >{v_c}$ reduces nutrient stress and hinders sinking of such heavier class, which entertain the possibility of bloom formation of heavier community on surface layer (Fig. 5c). This bloom now sustains for three days, and again due to the eventual stopping of wind event ($k_h<{k_h}^c,\,\langle u\rangle<{u_c},\,\langle v\rangle <{v_c}$), excessive phytoplankton biomass cannot spread over the larger domain and triggers water column instability. As a result, in the absence of a wind event, when there is no intense exchange of oceanic current ($\langle w\rangle \approx 0$), $k_v$ increases to restore water column stability but thermal stratification cannot let $k_v$ cross ${k_v}^c$, therefore on the very first day, species get transported to deeper layer (65th day, (Fig. 5d)), but eventually $k_v<{k_v}^c, \langle w\rangle <w_c$ triggers upwelling event, as a result of which amount of sinking biomass reduces (Fig. 5e), as wind strikes again on 70th day, the fact $k_h>{k_h}^c,\,\langle u\rangle>{u_c},\,\langle v\rangle >{v_c}$ keeps on providing ample supply of nutrient to existing phytoplankton community, and phytoplankton productivity gets enhanced. Under such conditions, extreme phytoplankton biomass floats away towards a more extensive domain (Fig. 4c), and water column stability, even in the presence of higher phytoplankton biomass, remains intact. Eventually, bloom occurs on the upper ocean surface (Fig. 5f). This also explains the fact why the bloom cycle of heavier species *“Skeletonema Costatum”* has been observed within a gap of certain days during wind mixing and thermally stratified period of 1990, 1991 (11–20 June, 17–25 June, 11–16 July, 13–17 July, 11–20 Aug, 11–23 Aug, 22–29 Sept, 2–8 Oct, 1–5 Mar, 10–17 Apr, 20–27 June, 29 July–2 Aug, 21–30 Sep) in Mikawa Bay, Japan^9^.

*We conclude the discussion by answering our final question, how does grazing control surface bloom dynamics of phytoplankton on two opposite sides of critical vertical eddy diffusivity* ${k_v}^c$  *for the almost calm aquatic environment?*

Grazing by herbivorous zooplankton plays a vital role in both scenarios, whether the bloom occurs on the surface layer or at a higher depth. For a fixed region with seasonal changes, the breeding and reproduction of herbivorous zooplankton generate differences in the overall population density of the grazing community, which controls the overall density of the phytoplankton community. In fact, at a particular time, this situation might arise with regional differences. As discussed in case SB3, whenever critical horizontal eddy diffusivity gets dominated by existing wind-induced horizontal eddy diffusivity in a turbulent flow, where the spatial average of intense horizontal velocity dominates its critical value, the chance of surface bloom development increases. Surface bloom under such circumstances can only be developed if and only if grazing impact remains negligible (as observed in SB3, SB4, Fig. 1e, 5c,f). In case, the grazing rate and assimilated grazing coefficient in the presence of low intra-specific competition among zooplankton classes exert a negative impact on the phytoplankton growth curve (Fig. 4d), formation of phytoplankton bloom (Fig. 4e) on surface water gets obstructed by higher zooplankton grazing on surface layer (Fig. 4f), even in a compatible environment in terms of horizontal eddy diffusivity and the spatial average of water velocity $\langle w\rangle$. It should be noted that higher grazing pressure cannot successfully dominate phytoplankton bloom development on the surface layer for a certain period for those aquatic environments, where an ample amount of nutrient gets supplied to the existing phytoplankton community in the presence of a ongoing upwelling event triggered by the condition ${k_v}<{k_v}^c$, $\langle w\rangle <w_c$ for system 2 and ${k_v}<{k_v}^c$ for system 1, which not only supplies sufficient nutrient but also transmit sinking phytoplankton biomass to surface layer. An abundance of nutrients and lack of sinking of phytoplankton class generates surface bloom even in the presence of higher grazing pressure for a certain amount of time, which has been observed in case SB2 (Fig. 1c), when compared to zooplankton biomass, nutrient density is so high that phytoplankton and zooplankton both grow together between approximately 70–110 days of the warmer season (Fig. 3e). Therefore, phytoplankton bloom gets developed on the surface layer (Fig. 1c) on the 115th day, while zooplankton is also abundant on the same day on the surface layer (Fig. 2b) due to high grazing pressure. Hence, if the nutrient supply remains much higher, then there exists a possibility of phytoplankton bloom for a certain amount of time, but, in case nutrient supply and zooplankton biomass both remain high together in a nutrient-rich zone during no wind mixing period and in the absence of any intense exchange of oceanic current ($\langle w\rangle \approx 0$), an absence of upwelling event does not transport deep nutrient-rich water and sinking phytoplankton biomass to the upper surface, instead $k_v>{k_v}^c$ shifts excessive phytoplankton biomass of buoyant community to a deeper layer. Therefore, in one direction, ample nutrient supply in nutrient-rich zones causes richness in phytoplankton biomass even in the presence of higher grazing pressure. On the other hand, as soon as excessive growth disturbs water column instability, surface biomass gets transported to the deeper layer. Whatever phytoplankton remains on the surface layer, higher grazing hinders its bloom formation on surface water (as observed in Fig. 4e). On the contrary, the availability of inadequate food sources in higher grazing pressure results in zooplankton bloom development on surface water (Fig. 4f). This has been numerically captured in Fig. 4, where $k_v>{k_v}^c$ along with high grazing pressure due to the abundance of zooplankton on the surface layer (Fig. 4f) obstruct phytoplankton growth (Fig. 4e) on surface water in a zone where both zooplankton and nutrient biomass are higher together (Fig. 4d). In fact, such a situation has been observed in a high nutrient zone of Gulf Alaska, where low diatom density is followed by an abundance in zooplankton^12^.

## Conclusion

The occurrence of phytoplankton bloom has always been a global phenomenon throughout all seasons for most aquatic ecosystems, including marine life of sea or ocean or stationary systems like lakes or ponds. Spatial and temporal contrasts in temperature, salinity, and chemical composition, along with differences in the distribution of biological agents, characterize the nature of aquatic systems. Depending on the quality of the water body and the characteristic of aquatic life, the nature of the phytoplankton community varies regionally and seasonally. Generally, there is a critical threshold value of water density for any aquatic system, whether it is freshwater or marine water. When this value is crossed, water becomes extremely stratified causing an imbalance in vertical mixing. This triggers vertical turbulent diffusivity $k_v$ and in the long run affects the distribution of the phytoplankton community. Habitually, variation in water temperature due to the lack of thermal stratification caused by low surface irradiance, wind-induced evaporation, and snowfall causes variation in water density. For example, the nature of thermal stratification governed by surface irradiance fluctuates with atmospheric variation and water density on the surface layer, which varies inversely with this thermal stratification and is directly proportional to salinity. The quality of water density stipulates the level of stratification. Generally, water quality varies with seasonal changes; hence level of stratification varies with climate change. As this happens, the stability of the water column gets affected, which influences the nature of horizontal ($k_h$) and vertical eddy diffusivity ($k_v$). This variation in diffusivity causes variation in mixing in a turbulent flow. On the other hand, changes in the direction of oceanic current with seasonal changes causes disturbances in water temperature. The surface water temperature remains relatively low during winter due to less thermal stratification, which causes an increment in the density of surface water. When this density crosses a critical threshold value, higher density on the surface layer makes the water column unstable. As a result, vertical turbulent mixing increases to transport excessive mass of plankton along with nutrients, which is required for photosynthesis, carried within the water column from the upper layer to the bottom layer until the column gets stabilized (Figs. 1g,h, 2d,h). It has long been recognized that the conditions of turbulence and mixing are critical factors for the growth and persistence of natural populations of phytoplankton in lakes and oceans^57–59^.

It should be noted that excluding eddy diffusivity $k_v,k_h$, the nature of water advection, being influenced by the spatial average of water velocity, also plays a vital role in the distribution of phytoplankton biomass. As far as systems like the lake, pond and calm oceanic medium (system 1) are concerned, usually spatial average of horizontal ($\langle u\rangle$, $\langle v\rangle$) and vertical ($\langle w\rangle$) water velocities remain zero in the absence of any externally induced artificial mixing. However, for a turbulent medium like a flowing river or rough marine ecosystem (system 2), external forces like seasonal tides, wind velocity, oceanic current, and sometimes natural calamity cause disturbances in the horizontal and vertical velocity distribution of water, hence the spatial average of horizontal and vertical water velocity, $\langle u\rangle \ne 0,\,\langle v\rangle \ne 0,\,\langle w\rangle \ne 0$, as a result of which distribution of planktonic system gets influenced.

The distribution of phytoplankton biomass and chances of bloom occurrence depend mainly on dominating nature of the control parameters $k_v,\,k_h$, $k_p,\,w_p$, $\langle u\rangle ,\,\langle v\rangle$, and $\langle w\rangle$ when the grazing impact is low. Among these control parameters, horizontal components ($k_h,\,k_p,\,\langle u\rangle ,\,\langle v\rangle$) trigger the chances of surface bloom, whereas vertical components ($k_v,\,w_p,\,\langle w\rangle$) enhance the chance of having bloom at higher depth. Dominating factors have specific critical values (determined from Routh Hurwitz stability criteria). The nature of the bloom and stability of the system depends on whether the dominating factors have crossed their corresponding critical threshold value in particular circumstances determined by the parameter values of the system. If each of the dominating factors is higher than the corresponding critical value, this determines the nature of phytoplankton bloom. However, once the bloom is developed either on the surface layer or at a higher depth, its sustainability depends on the system’s stability in particular circumstances determined by the parameter values of the system. As soon as the stability of the system is assured, if phytoplankton biomass remains higher at a certain depth $H_c$ or at bottom layer $L_z$ satisfying $H_c\,\text {or}\,L_z<D_c$ under the considered circumstances, then this higher biomass will trigger bloom initiation at that depth $H_c$ or at the bottom layer $L_z$ of shallow water being influenced by the stability of the system, which has been observed for all above considered cases (SB1-SB4, BHD1, and BHD2).

Therefore, for any aquatic environment like a still lake, pond, or almost calm marine environment (system 1), we may conclude the following facts regarding phytoplankton bloom dynamics in the presence of low grazing impact,

(i) surface bloom of buoyant species will mostly occur during a warmer season without any artificial or wind-induced mixing. Even in the presence of artificial mixing, a surface bloom of buoyant species can occur when vertical eddy diffusion $k_v<{k_v}^c$ cannot transport all phytoplankton biomass to higher depth (Case SB1, (SI Table 1)). This situation is observed when multi-species exist in a lake, pond, or almost calm marine environment, where one community is buoyant, and some have a higher sinking tendency. Then, during the warmer season, there will be a higher chance of having surface bloom of buoyant species under the circumstances where existing vertical eddy diffusion remains sufficiently low to be dominated by its corresponding critical turbulence.
(ii) But, if artificial mixing or cold weather influences the aquatic environment of the lake, pond, or almost stationary aquatic flow and affects the stability of the water column, then most bloom will occur at higher depths in the presence of low grazing depending on two facts (a) dominance of ${k_v}^c$ by existing vertical eddy diffusivity $k_v$ (b) the depth ($H_c$ or $L_z$) at which sinking species gets stuck, is less than the critical depth ($D_c$) (cases BHD1, BHD2, SB1, (SI Table 1).

As far as turbulent flow is concerned (system 2), we obtain the following facts regarding bloom formation in the presence of low grazing influence,

(i) when wind-induced horizontal turbulent diffusion $k_h$ dominates vertical turbulence $k_v$ mostly during a thermally stratified period and the spatial average of horizontal water velocity dominates corresponding vertical component as well influenced by the act of surface wind event. The chance of surface development, irrespective of the nature of the existing community, increases provided the fact that horizontal eddy diffusivity and the spatial average of horizontal water velocity dominate their corresponding critical values ($k_h>{k_h}^c,\langle u\rangle>u_c,\langle v\rangle >v_c$, cases SB3, SB4, 1e, 5d,f (SI Table 2)). If the system is in motion, and the horizontal mixing rate dominates, then the surface bloom of any species can also occur in turbulent flow under the influence of proper wind events. Mostly, when the system is almost calm during the warmer season, then surface bloom of buoyant species will occur,
(ii) as winter season passes by and spring approaches during no wind mixing period, if thermal stratification results in $k_v<{k_v}^c$ and initiates an upwelling event, then the possibility of bloom formation on the upper ocean or surface layer increases, as observed in the cases BHD1, BHD2 for system 2 (Figs. 2c, 2g),
(iii) if in the presence of artificial mixing or during cold weather, when existing vertical turbulent diffusivity and the spatial average of water velocity dominate corresponding critical values in system 2 ($k_v>{k_v}^c,\langle w\rangle >w_c$, cases BHD1, BHD2, (SI Table 2)), then in the presence of low grazing pressure, sinking phytoplankton biomass will initiate the chance of bloom formation at a higher depth ($H_c$ or $L_z$) at which sinking species gets when $H_c \,\text {or}\,L_z<D_c$.

Different phytoplankton communities possess several behavioral traits, like the nature of sinking or swimming speed and motility. In lakes, many phytoplankton classes coexist together. Depending on nature of zooplankton grazing and how environmental factors are influencing nature of phytoplankton productivity, phytoplankton bloom initiation is dominated by the class capable enough to exist and grow beyond all hindrances. As climate variation or regional shift occurs, the communities vary. These properties also vary, which play a vital role in phytoplankton bloom at several layers during several periods. When it comes to the turbulent marine ecosystem, spring and summer surface bloom are very common, but winter surface bloom is rarely observed. However, if winter bloom is observed, they are primarily found in the deep ocean or sea. Therefore, the nature of bloom formation varies with seasonal changes, which can be related to generated spatiotemporal contrast in essential components of water body of aquatic medium, for example, eddy diffusivity, water stratification etc. influenced by elementary factors like temperature, evaporation, rainfall, snowfall, ice melting, etc., which vary with seasonal changes.

### Supplementary Information

Supplementary Information.

## Data Availability

All data generated or analysed during this study are included in this published article (and its supplementary information file).
