# Supplementary material for: Impact of critical eddy diffusivity on seasonal bloom dynamics of Phytoplankton in a global set of aquatic environments
Source: Sci Rep. 2023 Oct 10;13:17141. doi: 10.1038/s41598-023-43745-z (PMC10564959; doi:10.1038/s41598-023-43745-z)
Supplement: Supplementary file 1 — Supplementary Information. [file 41598_2023_43745_MOESM1_ESM.pdf]

Impact of critical eddy diffusivity on seasonal bloom dynamics of phytoplankton in a global set of aquatic environments (Supplementary Information)

Arpita Mondal[1] **and Sandip Banerjee**[2,*]

[1,2]Department of Mathematics, Indian Institute of Technology Roorkee, Roorkee 247667, Uttarakhand, India
[*]Corresponding author

ABSTRACT

Derivation of closure system and calculation of critical depth are shown in this section. The parameter sets for each figures, calculation of critical depth, and tables for system 1 and system 2 are also given here.

1 Formation of Closure Model

We know,

$$S_1(N,P,Z) = -\mu_p\, P + (m_P + r + s + k_2) + (1-a_z)g_zPZ + (\tau_z + m_zZ)Z, \text{ where } \mu_p = g(I)\, F(N,P), \tag{1}$$

$$S_2(N,P,Z) = \mu_p\, P - (m_P + r + s + k_2)\, P - g_zP\, Z, \tag{2}$$

$$S_3(N,P,Z) = a_zg_zPZ - (\tau_z + m_zZ)\, Z, \tag{3}$$

$$N(r,t) = N_0(r,t) + N^{'}(r,t),\; P(r,t) = P_0(r,t) + P'(r,t) \text{ and } Z(r,t) = Z_0(r,t) + Z'(r,t). \tag{4}$$

Substituting (4) in (2), we obtain,

$$S_1(N,P,Z) = -\mu_p\, P + (m_P + r + s + k_2)P + (1-a_z)g_zPZ + (\tau_z + m_zZ)Z$$

$$\Rightarrow S_1(N,P,Z) = -g(I)F(N,P)\, P + (m_P + r + s + k_2)P + (1-a_z)g_zPZ + (\tau_z + m_zZ)Z$$

$$\Rightarrow S_1(N_0+N', P_0+P', Z_0+Z') = -g(I)\frac{N_0+N'}{K+N_0+N'}(P_0+P') + (m_P + r + s + k_2)(P_0+P')$$
$$+ (1-a_z)g_z(P_0+P')(Z_0+Z') + \tau_z(Z_0+Z') + m_z(Z_0+Z')^2$$

$$\Rightarrow S_1(N_0+N', P_0+P', Z_0+Z') = -g(I)\frac{N_0P_0+N'P_0+N_0P'+N'P'}{K+N_0+N'} + (m_P + r + s + k_2)(P_0+P')$$
$$+ (1-a_z)g_z(P_0Z_0+P_0Z'+P'Z_0+P'Z') + \tau_z(Z_0+Z') + m_z({Z_0}^2 + Z'^2 + 2Z_0Z')$$

$$\Rightarrow S_1(N_0+N', P_0+P', Z_0+Z') = -g(I)\frac{N_0P_0+N'P_0+N_0P'+N'P'}{K+N_0}(1+\frac{N'}{K+N_0})^{-1}$$
$$+ (m_P + r + s + k_2)(P_0+P') + (1-a_z)g_z(P_0Z_0+P_0Z'+P'Z_0+P'Z') + \tau_z(Z_0+Z')$$
$$+ m_z({Z_0}^2 + Z'^2 + 2Z_0Z')$$

$$\Rightarrow S_1(N_0+N', P_0+P', Z_0+Z') = -g(I)\frac{N_0P_0+N'P_0+N_0P'+N'P'}{K+N_0}(1-\frac{N'}{K+N_0}+\frac{N'^2}{(K+N_0)^2})$$
$$+ (m_P + r + s + k_2)(P_0+P') + (1-a_z)g_z(P_0Z_0+P_0Z'+P'Z_0+P'Z') + \tau_z(Z_0+Z')$$
$$+ m_z({Z_0}^2 + Z'^2 + 2Z_0Z')$$

$$\Rightarrow S_1(N_0+N', P_0+P', Z_0+Z') = -\frac{g(I)}{K+N_0}\left(N_0P_0+N'P_0+N_0P'+N'P'\right)$$
$$-\frac{g(I)}{(K+N_0)^2}\left(N_0P_0N'+N'^2P_0+N_0P'N'\right) + \frac{g(I)}{(K+N_0)^3}\left(N_0P_0N'^2\right) + (m_P + r + s + k_2)(P_0+P')$$
$$+ (1-a_z)g_z(P_0Z_0+P_0Z'+P'Z_0+P'Z') + \tau_z(Z_0+Z') + m_z({Z_0}^2 + Z'^2 + 2Z_0Z').$$

Taking average on both sides and using properties of Reynold's average along with $< N^{'} >$=0, $< P' >$=0, $< Z' >$=0, we obtain

$$\Rightarrow \langle S_1(N_0+N',P_0+P',Z_0+Z')\rangle = -\frac{g(I)}{K+N_0}\left(N_0P_0+\langle N'P'\rangle\right) - \frac{g(I)}{(K+N_0)^2}\left(\langle N'^2\rangle P_0 + N_0\langle P'N'\rangle\right)$$
$$+\frac{g(I)}{(K+N_0)^3}\left(N_0P_0\langle N'^2\rangle\right) + (m_P+r+s+k_2)P_0 + (1-a_z)g_z(P_0Z_0+\langle P'Z'\rangle) + \tau_z Z_0 + m_z({Z_0}^2+\langle Z'^2\rangle)$$
$$\Rightarrow \langle S_1(N_0+N',P_0+P',Z_0+Z')\rangle = -\frac{g(I)N_0P_0}{K+N_0} - \frac{Kg(I)\langle P'N'\rangle}{(K+N_0)^2} + \frac{Kg(I)P_0\langle N'^2\rangle}{(K+N_0)^2} + (m_P+r+s+k_2)P_0$$
$$+(1-a_z)g_z(P_0Z_0+\langle P'Z'\rangle) + \tau_z Z_0 + m_z({Z_0}^2+\langle Z'^2\rangle), \tag{5}$$

Similarly we obtain,

$$\langle S_2(N_0+N',P_0+P',Z_0+Z')\rangle = \frac{g(I)N_0P_0}{K+N_0} + \frac{Kg(I)\langle P'N'\rangle}{(K+N_0)^2} - \frac{Kg(I)P_0\langle N'^2\rangle}{(K+N_0)^2} - (m_P+r+s+k_2)P_0$$
$$-g_z(P_0Z_0+\langle P'Z'\rangle), \tag{6}$$

$$\langle S_3(N_0+N',P_0+P',Z_0+Z')\rangle = a_z g_z(P_0Z_0+\langle P'Z'\rangle) - \tau_z Z_0 - m_z({Z_0}^2+\langle Z'^2\rangle), \tag{7}$$

$$2\langle P'S_2(N_0+N',P_0+P',Z_0+Z')\rangle = 2g(I)\left(\frac{N_0\langle P'^2\rangle}{(K+N_0)} + \frac{kP_0\langle N'P'\rangle}{(K+N_0)^2}\right) - 2(m_P+r+s+k_2)\langle P'^2\rangle$$
$$-2g_z\left(P_0\langle P'Z'\rangle + Z_0\langle P'^2\rangle\right), \tag{8}$$

$$2\langle Z'S_3(N_0+N',P_0+P',Z_0+Z')\rangle = 2a_z g_z\left(P_0\langle Z'^2\rangle + Z_0\langle P'Z'\rangle\right) - 2\tau_z\langle Z'^2\rangle - 4m_z Z_0\langle Z'^2\rangle, \tag{9}$$

$$2\langle N'S_1(N_0+N',P_0+P',Z_0+Z')\rangle = -2g(I)\left(\frac{N_0\langle N'P'\rangle}{(K+N_0)} + \frac{kP_0\langle N'^2\rangle}{(K+N_0)^2}\right) + 2(m_P+r+s+k_2)\langle N'P'\rangle$$
$$+2(1-a_z)g_z\left(P_0\langle N'Z'\rangle + Z_0\langle N'P'\rangle\right) + 2\tau_z\langle N'Z'\rangle + 4m_z Z_0\langle N'Z'\rangle, \tag{10}$$

$$\langle P'S_3(N_0+N',P_0+P',Z_0+Z')\rangle + \langle Z'S_2(N_0+N',P_0+P',Z_0+Z')\rangle = g(I)\left(\frac{N_0\langle P'Z'\rangle}{(K+N_0)} + \frac{kP_0\langle N'Z'\rangle}{(K+N_0)^2}\right)$$
$$-(m_P+r+s+k_2)\langle P'Z'\rangle - g_z\left(P_0\langle Z'^2\rangle + (Z_0-aP_0)\langle P'Z'\rangle - aZ_0\langle P'^2\rangle\right) - \tau_z\langle P'Z'\rangle - 2m_z Z_0\langle P'Z'\rangle, \tag{11}$$

$$\langle P'S_1(N_0+N',P_0+P',Z_0+Z')\rangle + \langle N'S_2(N_0+N',P_0+P',Z_0+Z')\rangle$$
$$= g(I)\left(\frac{N_0(\langle N'P'\rangle - \langle P'^2\rangle)}{(K+N_0)} + \frac{kP_0(\langle N'^2\rangle - \langle N'P'\rangle)}{(K+N_0)^2}\right) + (m_P+r+s+k_2)(\langle P'^2\rangle - \langle N'P'\rangle)$$
$$+g_z\left(P_0((1-a)\langle P'Z'\rangle - \langle N'Z'\rangle) + Z_0((1-a)\langle P'^2\rangle - \langle N'P'\rangle)\right) + \tau_z\langle P'Z'\rangle + 2m_z Z_0\langle P'Z'\rangle, \tag{12}$$

$$\langle Z'S_1(N_0+N',P_0+P',Z_0+Z')\rangle + \langle N'S_3(N_0+N',P_0+P',Z_0+Z')\rangle = -g(I)\left(\frac{N_0\langle P'Z'\rangle}{(K+N_0)} + \frac{kP_0\langle N'Z'\rangle}{(K+N_0)^2}\right)$$
$$+(m_P+r+s+k_2)\langle P'Z'\rangle + \tau_z\left(\langle Z'^2\rangle - \langle N'Z'\rangle\right) + 2m_z Z_0\left(\langle Z'^2\rangle - \langle N'Z'\rangle\right)$$
$$g_z\left(P_0((1-a)\langle Z'^2\rangle + a\langle N'Z'\rangle + Z_0((1-a)\langle P'Z'\rangle + a\langle N'P'\rangle)\right). \tag{13}$$

Now summing (5), (6), (7), we obtain $\sum_{i=1}^{3}\langle S_i\rangle = 0$ and sum of (8), (9), (10) and twice of (11), (12), (13) gives zero.

1.1 Functional form of $f_i(V)$ for $i = 1,2,...,7$

$$f_1(U) = \frac{r(I)(1-p_0-z_0)p_0}{k+1-p_0-z_0} + \frac{kr(I)\beta\langle n'p'\rangle}{(k+1-p_0-z_0)^2} - \frac{Kr(I)p_0\beta\langle n'^2\rangle}{(k+1-p_0-z_0)^3} - \frac{(m_P+r+s+k_2)}{V_m}p_0$$
$$-\frac{g_zA}{V_m}(p_0z_0+\beta\langle p'z'\rangle),$$

$$f_2(U) = \frac{a_zg_zA}{V_m}(p_0z_0+\beta\langle p'z'\rangle) - \frac{\tau_z}{V_m}z_0 - \frac{m_zA}{V_m}({z_0}^2+\beta\langle z'^2\rangle),$$
$$f_3(U) = 2r(I)\left(\frac{(1-p_0-z_0)\langle p'^2\rangle}{(K+1-p_0-z_0)} + \frac{kp_0\langle n'p'\rangle}{(K+1-p_0-z_0)^2}\right) - 2\frac{(m_P+r+s+k_2)}{V_m}\langle p'^2\rangle$$
$$-2\frac{g_zA}{V_m}\left(p_0\langle p'z'\rangle + z_0\langle p'^2\rangle\right),$$

$$f_4(U) = 2\frac{a_zg_zA}{V_m}\left(p_0\langle z'^2\rangle + z_0\langle p'z'\rangle\right) - 2\frac{\tau_z}{V_m}\langle z'^2\rangle - 4\frac{m_zA}{V_m}z_0\langle z'^2\rangle,$$

$$f_5(U) = -2r(I)\left(\frac{(1-p_)-z_0)\langle n'p'\rangle}{(k+1-p_0-z_0)} + \frac{kp_0\langle n'^2\rangle}{(K+1-p_0-z_0)^2}\right) + 2\frac{(m_P+r+s+k_2)}{V_m}\langle n'p'\rangle$$
$$+2\frac{(1-a_z)g_zA}{V_m}\left(p_0\frac{1}{2}(1-\langle p'^2\rangle-\langle n'^2\rangle-\langle z'^2\rangle-2\langle n'p'\rangle-2\langle p'z'\rangle)+z_0\langle n'p'\rangle\right)$$
$$+2\frac{\tau_z}{V_m}\frac{1}{2}(1-\langle p'^2\rangle-\langle n'^2\rangle-\langle z'^2\rangle-2\langle n'p'\rangle-2\langle p'z'\rangle)$$
$$+4\frac{m_zA}{V_m}z_0\frac{1}{2}(1-\langle p'^2\rangle-\langle n'^2\rangle-\langle z'^2\rangle-2\langle n'p'\rangle-2\langle p'z'\rangle),$$

$$f_6(U) = r(I)\left(\frac{n_0\langle p'z'\rangle}{(k+1-p_0-z_0)} + \frac{kp_0(1-\langle p'^2\rangle-\langle n'^2\rangle-\langle z'^2\rangle-2\langle n'p'\rangle-2\langle p'z'\rangle)}{2(k+1-p_0-z_0)^2}\right)$$
$$-\frac{(m_P+r+s+k_2)}{V_m}\langle p'z'\rangle - \frac{g_zA}{V_m}\left(p_0\langle z'^2\rangle+(z_0-ap_0)\langle p'z'\rangle-az_0\langle p'^2\rangle\right) - \frac{\tau_z}{V_m}\langle p'z'\rangle - 2\frac{m_zA}{V_m}z_0\langle p'z'\rangle,$$

$$f_7(U) = r(I)\left(\frac{(1-p_0-z_0)(\langle n'p'\rangle-\langle p'^2\rangle)}{(K+1-p_0-z_0)} + \frac{kp_0(\langle n'^2\rangle-\langle n'p'\rangle)}{(k+1-p_0-z_0)^2}\right) + \frac{(m_P+r+s+k_2)}{V_m}(\langle p'^2\rangle-\langle n'p'\rangle)$$
$$+\frac{g_zA}{V_m}\left(p_0((1-a)\langle p'z'\rangle-\frac{1}{2}(1-\langle p'^2\rangle-\langle n'^2\rangle-\langle z'^2\rangle-2\langle n'p'\rangle-2\langle p'z'\rangle))+z_0((1-a)\langle p'^2\rangle-\langle n'p'\rangle)\right)$$
$$+\frac{\tau_z}{V_m}\langle p'z'\rangle + 2\frac{m_zA}{V_m}z_0\langle p'z'\rangle.$$

2 Calculation of Critical Depth (D_c)

For the considered cases BHD1, BHD2, when bloom occurs at higher depth during winter season, surface irradiance is $I_0 = 160\ W/m^2$, therefore integrated surface PAR,

$$\sum E_0 = \frac{I_0 \times 4.6 \times 3600 \times 24}{10^6} \text{ mmol photon } m^{-2}\ day^{-1},$$

$\kappa = 0.02\ m^{-1}$, therefore,

$$D_c = \frac{\sum E_0}{3.78\kappa} \approx 841 \text{ meters for cases BHD1, BHD2.} \tag{14}$$

3 Parameter Set

Set of all parameter values for this simulation are as follows:

3.1 Case: SB1 (System 1)

$\kappa = 0.04, I_0 = 320, k_p = 5, H = 50, w_p = 0.012, m_p = 0.07, r = 0, s = 0, k_2 = 0.001, m_z = 0.2$ $\tau_z = 0.050112, < u >= 0, < v >= 0, < w >= 0, k_h = 0.01, k_v = 4(k_v << {k_v}^c \approx 953.762, \sigma_3 = 2), k_v = 140(k_v > {k_v}^c = 105.975, \sigma_3 = 6), A = 1, K = 1.6, g_z = 0.4, a_z = 0.3, k01 = 0, w01 = 0, k02 = 0, w02 = 0,$ $\beta = 5, \alpha = 0.015, V_m = 2.5, H = 10, l = 60, \sigma_1 = \sigma_2 = 0.01.$

3.2 Case:SB2 (System 1)

$\kappa = 0.08, I_0 = 220, k_p = 0.001, H = 20, w_p = 2.7, m_p = 0.07, r = 0, s = 0, k_2 = 0.001,$ $m_z = 0.3, \tau_z = 0.050112,$ $\langle u \rangle = 0, \langle v \rangle = 0, \langle w \rangle = 0,$ $k_h = 0.01, k_v = 20$ (while $k_v < {k_v}^c = 667.545$), $A = 1, K = 1.6, g_z = 0.8, a_z = 0.4, \beta = 5, \alpha = 0.015$ $V_m = 2.5, H = 10, l = 60, \sigma_1 = \sigma_2 = 0.01, \sigma_3 = 1.$

3.3 Case: SB3 (System 2)

$\kappa = 0.01, I_0 = 320, k_p = 0.01, H = 50, w_p = 6, m_p = 0.07, r = 0, s = 0.04, k_2 = 0.001, m_z = 0.2$ $\tau_z = 0.050112, < u >= 14, < v >= 14$ $(< u >>< u >^c = 6.30452, < v >>< v >^c = 6.30452), < w >= 0.001, k_h = 140$ $(k_h > {k_h}^c = 62.0452, \sigma_1 = \sigma_2 = 1, \sigma_3 = 0.1), k_h = 140$ $(k_h < {k_h}^c = 575.221, \sigma_3 = 3.2, \sigma_1 = \sigma - 2 = 0.5, k_v = 10(< {k_v}^c = 775.037), \langle u \rangle = 10(< u_c = 31.7611), \langle v \rangle = 10(< v_c = 31.7611), \langle w \rangle = 2(< w_c = 42.802), k_v = 10, A = 1, K = 1.6, g_z = 0.4, a_z = 0.3, k01 = 0, w01 = 0, k02 = 0, w02 = 0,$ $\beta = 5, \alpha = 0.015, V_m = 2.4, H = 10, l = 60.$ Fig (1e) $(< u >= 10 < u_c = 31.7611, < v >= 10 < v_c = 31.7611, < w >= 2 < w_c = 42.802,$ $k_h = 140 < {k_h}^c = 575.221, k_v = 10 < {k_v}^c = 775.037.)$

3.4 Case: SB4 (System 2)

$\kappa = 0.01, I_0 = 320, k_p = 0.01, H = 50, w_p = 6, m_p = 0.07, r = 0, s = 0.04, k_2 = 0.001, m_z = 0.2$ $\tau_z = 0.050112, < u >= 5, < v >= 5$ $(< u ><< u >^c, < v ><< v >^c), < u >= 5, < v >= 5$ $(< u ><< u >^c = 9.50762, < v ><< v >^c = 9.50762), < w >= 0.001 < w_c = 8.68294, k_h = 90$ $(k_h < {k_h}^c = 135.076, \sigma_3 = 3, k_v = 10 < {k_v}^c = 370.061), k_h = 140$ $(k_h > {k_h}^c), k_v = 10, A = 1, K = 1.6, g_z = 0.4, a_z = 0.3, k01 = 0, w01 = 0, k02 = 0, w02 = 0,$ $\beta = 5, \alpha = 0.015, V_m = 2.4, H = 10, l = 60, \sigma_1 = 1, \sigma_2 = 1.$ Figure (1f)-$\langle u \rangle = 5 < u_c = 9.50762,$ $\langle v \rangle = 5 < v_c = 9.50762, k_v = 10 < {k_v}^c = 370.61,$ $< w >= 0.001 < w_c = 18.0315, k_h = 90 < {k_v}^c = 135.076$, Figure (2a-2b, 2d-2e$< u >= 5, < v >= 5$ $(< u ><< u >^c = 9.34097, < v ><< v >^c = 9.34097), k_h = 90$ $(k_h < {k_h}^c = 133.41), \sigma_3 = 6)$, Figure (2c $< u >= 10, < v >= 10$ $(< u >>< u >^c = 4.39253, < v >>< v >^c = 4.39253), k_h = 140$ $(k_h > {k_h}^c = 83.9253), \sigma_3 = 0.6$, Figure (2f $< u >= 10, < v >= 10$ $(< u >>< u >^c = 4.33667, < v >>< v >^c = 4.33667), k_h = 140$ $(k_h > {k_h}^c = 83.3667), \sigma_3 = 0.1.$

3.5 Case: BHD1 (System 1)

$\kappa = 0.02, I_0 = 160, k_p = 0.01, H = 50, w_p = 5, m_p = 0.07, r = 0, s = 0, k_2 = 0.001, m_z = 0.2$ $\tau_z = 0.050112, < u >= 0, < v >= 0, < w >= 0, k_v = 200(k_v > {k_v}^c = 172.621, k_h = 4, \sigma_1 = 0.01, \sigma_2 = 0.01, \sigma_3 = 6), k_v = 90(k_v < {k_v}^c = 2734.91, k_h = 10, \sigma_1 = 0.1, \sigma_2 = 0.1, \sigma_3 = 1.5), A = 1, K = 1, g_z = 0.4, a_z = 0.3, k01 = 0, w01 = 0, k02 = 0, w02 = 0,$ $\beta = 5, \alpha = 0.015, V_m = 1, H = 10, l = 60, , .$

3.6 Case: BHD1 (System 2)

$\kappa = 0.02, I_0 = 160, k_p = 0.01, H = 50, w_p = 5, m_p = 0.07, r = 0, s = 0, k_2 = 0.001, m_z = 0.2$ $\tau_z = 0.050112, < u >= 0.01, < v >= 0.01, < w >= 10(< w >> w_c = 8.26176), < w >= 10(< w >< w_c = 29.8302), k_h = 10, k_v = 90(k_v > {k_v}^c = 72.6176, \sigma_1 = 0.01, \sigma_2 = 0.01, \sigma_3 = 6), k_v = 90(k_v < {k_v}^c = 486.604, \sigma_1 = 0.01, \sigma_2 = 0.01, \sigma_3 = 3), A = 1, K = 1, g_z = 0.4, a_z = 0.3, k01 = 0, w01 = 0, k02 = 0, w02 = 0,$ $\beta = 5, \alpha = 0.015, V_m = 1, H = 10, l = 60, \sigma_1 = 0.01, \sigma_2 = 0.01.$

3.7 Case: BHD2 (System 1)

$\kappa = 0.02, I_0 = 160, k_p = 5, H = 50, w_p = 0.012, m_p = 0.07, r = 0, s = 0, k_2 = 0.001, m_z = 0.2$ $\tau_z = 0.050112, < u >= 0, < v >= 0, < w >= 0, k_h = 1, k_v = 250(k_v > {k_v}^c = 244.869, \sigma_3 = 5), k_v = 120(k_v < {k_v}^c = 6121.46, \sigma_3 = 1), A = 1, K = 1, g_z = 0.4, a_z = 0.3, k01 = 0, w01 = 0, k02 = 0, w02 = 0,$ $\beta = 5, \alpha = 0.015, V_m = 1, H = 10, l = 60, \sigma_1 = \sigma_2 = 0.01.$

3.8 Case: BHD2 (System 2)

$\kappa = 0.02, I_0 = 160, k_p = 5, H = 50, w_p = 0.012, m_p = 0.07, r = 0, s = 0, k_2 = 0.001, m_z = 0.2$ $\tau_z = 0.050112, < u >= 0.01, < v >= 0.01, < w >= 10(< w >> w_c = 5.00477, \sigma_3 = 6), < w >= 6(< w >= 10 < w_c = 30.8376, \sigma_3 = 2.8), k_h = 1, k_v = 120(k_v > {k_v}^c = 70.0477, \sigma_3 = 6), k_v = 120(k_v < {k_v}^c = 566.519, \sigma_3 = 2.8), A = 1, K = 1, g_z = 0.4, a_z = 0.3, k01 = 0, w01 = 0, k02 = 0, w02 = 0,$ $\beta = 5, \alpha = 0.015, V_m = 1, H = 10, l = 60, \sigma_1 = \sigma_2 = 0.01.$

3.9 Higher Grazing

$\kappa = 0.01, I_0 = 320, k_p = 5, H = 50, w_p = 0.012, m_p = 0.15, r = 0, s = 0, k_2 = 0.001, m_z = 0.0, \tau_z = 0.050112, \langle u\rangle = 0.4, \langle v\rangle = 0.4$ (while $\langle u\rangle > u_c = 0.397843, \langle v\rangle > v_c = 0.397843$) , $\langle w\rangle = 0.001, k_h = 10$ (while $k_h > {k_h}^c = 9.97608$) $k_v = 0.2 (> {k_v}^c = 0.112436), A = 1, K = 1.6, g_z = 1, a_z = 0.5, \beta = 5, \alpha = 0.015, V_m = 1.9, H = 10, l = 60, \sigma_1 = \sigma_2 = 0.902, \sigma_3 = 4.$

4 Tables

Case	Nature of Dominating Class	Relation between velocity terms	Relation between diffusivity terms	Overall dominating quantities	Relation between existing and critical value	Relation between H_c/L_z and D_c	Nature of bloom
BHD1	Heavier ($w_p > k_p$)	$< w >= 0$ $< u >= 0$ $< v >= 0$	$k_v > k_h$	k_v	${k_v}^c \approx 172.621 < k_v = 2 = 300$	$L_z = 60$ $D_c = 841$	Bloom at higher depth (L_z)
BHD2	Buoyant ($k_p > w_p$)	$< w >= 0$ $< u >= 0$ $< v >= 0$	$k_v > k_h$	k_v	${k_v}^c \approx 244.869 < k_v = 250$	$L_z = 60$ $D_c = 841$	Bloom at higher depth (L_z)
SB1	Buoyant ($k_p > w_p$)	$< w >= 0$ $< u >= 0$ $< v >= 0$	$k_v > k_h$	k_v	${k_v}^c \approx 105.975 < k_v = 140$	$L_z = 60$ $D_c = 841$	Bloom at L_z
SB2	Heavier ($w_p > k_p$)	$< w >= 0$ $< u >= 0$ $< v >= 0$	$k_v > k_h$	k_v	${k_v}^c \approx 667.545 > k_v = 20$		Surface bloom

Supplementary table 1. Dominating quantity and nature of bloom: System 1

Case	Nature of Dominating Class	Relation between velocity terms	Relation between diffusivity terms	Overall dominating quantities	Relation between existing and critical value	Relation between H_c/L_z and D_c	Nature of bloom
BHD1	Heavier ($w_p > k_p$)	$\langle w\rangle > \langle u\rangle, \langle v\rangle$	$k_v > k_h$	$k_v, \langle w\rangle$	$w_c \approx 8.26176 < \langle w\rangle = 10$ ${k_v}^c \approx 72.6176 < k_v = 90$	$L_z = 60$ $D_c = 841$	Bloom at higher depth (L_z)
BHD2	Buoyant ($k_p > w_p$)	$\langle w\rangle > \langle u\rangle, \langle v\rangle$	$k_v > k_h$	$k_v, \langle w\rangle$	$w_c \approx 5.00477 < \langle w\rangle = 10$ ${k_v}^c \approx 70.0477 < k_v = 120$	$L_z = 60$ $D_c = 841$	Bloom at higher depth (L_z)
SB3	Buoyant ($k_p > w_p$)	$\langle u\rangle, \langle v\rangle > \langle w\rangle$	$k_h > k_v$	k_h $\langle u\rangle, \langle v\rangle$	$u_c \approx 6.30452 < \langle u\rangle = 14$ $v_c \approx 6.30452 < \langle v\rangle = 14$ ${k_h}^c \approx 63.0452 < k_h = 140$		Surface bloom
SB4	Heavier ($k_p > w_p$)	$\langle u\rangle, \langle v\rangle > \langle w\rangle$	$k_h > k_v$	k_h $\langle u\rangle, \langle v\rangle$	$u_c \approx 4.39253 < \langle u\rangle = 10$ $v_c \approx 4.39253 < \langle v\rangle = 10$ ${k_h}^c \approx 83.9253 < k_h = 140$		Surface bloom

Supplementary table 2. Dominating quantity and nature of bloom: System 2
